# Research on the impact of ecological additives on selected parameters and the microbiological distribution of marine engine oil

**DOI:** 10.1038/s41598-025-28841-6

**Published:** 2025-12-16

**Authors:** Rafał Krakowski

**Affiliations:** Faculty of Marine Engineering, Gdynia Maritime University, Morska 81-87, Gdynia, 81-225 Poland

**Keywords:** Engine oil, Operating parameters, Oil properties, Effective microorganisms, Silver compounds, Biotechnology, Engineering, Environmental sciences, Microbiology

## Abstract

The article presents the impact of ecological additives on selected parameters and the microbiological distribution of marine engine oil (Marinol). With the development of industry and the automotive sector in general, it has become apparent that the problem of microbial contamination is still relevant and is becoming increasingly widespread. The author decided to investigate the impact of additives, namely silver and effective microorganisms with a 2% percentage share in marine engine oil (Marinol), on selected parameters and on slowing down the growth of bacteria and fungi. The article presents the impact of additives on selected engine oil parameters, including flash point, water content, acid and base numbers, density and kinematic viscosity. In addition, the oil was also tested for the effect of additives on the inhibition of microbial decomposition in the tested oil. It was found that the best additive with a positive effect on engine oil parameters and microbial decomposition was a silver solution and effective microorganisms in the form of ceramics. The addition of effective microorganisms in liquid form and silver solution to used oil resulted in an increase in temperature of approximately 1 °C compared to the ignition temperature of oil without additives. For the addition of colloidal nanosilver, a temperature of 225.22 °C was obtained. For used oil, the water content increased to 0.16% from 0.1% for new oil. The addition of liquid effective microorganisms caused the largest increase in water content to 0.58%, while EM in the form of ceramics did not contribute to the increase in water content due to its composition. The addition of silver solution contributed to an increase in water content to 0.47% and colloidal silver to 0.53%. The base number of used motor oil without additives decreased from 11.82 mgKOH/g for fresh oil to 11.52 mgKOH/g. After the application of ceramic effective microorganisms, a lower base number was obtained compared to oil without additives, which is 11.34 mgKOH/g, while liquid effective microorganisms reduced the base number the most, to 11.27 mgKOH/g. Each of the additives caused a decrease in bacteria and fungi compared to used oil without additives. The use of these additives is an original solution that has a positive effect on the microbial degradation process, while maintaining the original performance parameters of the engine oil. In the next stage, it would be desirable to confirm the results obtained under actual operating conditions.

## Introduction

 The ongoing development of internal combustion engines means that the oils used in them are operating under increasingly demanding conditions. This is due to the increase in unit power while simultaneously reducing the efficiency of lubrication systems and extending the periods between lubricant changes. Design changes to drive units and modifications to fuel composition are largely a consequence of growing environmental protection requirements^1,2^.

As a result, engine manufacturers are forced to implement innovative technologies that enable them to adapt to increasingly stringent requirements related to the reduction of atmospheric emissions. However, these challenges affect not only engine designers, but also the fuel sector and lubricant manufacturers^3–5^.

When a diesel engine is operated correctly, the main factors that deteriorate its technical condition are various types of mechanical and chemical contaminants. These include both solid particles and corrosive substances. Among the many contaminants that enter the drive system from outside or are generated during the operation of the unit, the most adverse effect is caused by contaminants present in the fuel and in the engine oil circulating in the lubrication system. Therefore, a diesel engine should be fuelled with fuel containing as few harmful additives as possible, and engine oil should be changed when the accumulation of contaminants reaches excessive levels. Neglect in this regard inevitably leads to accelerated wear and tear of the drive unit components and various malfunctions in its operation^6–8^.

During the use of engine oil, changes in its physicochemical properties occur, referred to as the ageing process. Phenomena such as soot accumulation, gel and sludge formation, and the creation of polymer structures leading to increased viscosity are particularly important, as they directly affect the safety and economy of engine operation. It is worth noting that detailed chemical analyses indicate that these sludges are increasingly not the result of oil oxidation, as there is no significant increase in the content of carbonyl compounds or the presence of nitrogen compounds such as nitro derivatives. This suggests a different mechanism of their formation, which may be related to the changing composition of modern oils and fuels^9–11^.

The dynamic development of the automotive industry, as well as the aviation and maritime sectors, is causing an increase in demand for hydrocarbon fuels. As a result, their microbial contamination is becoming a significant problem, affecting not only motor, aviation and marine fuels, but also engine oils, lubricants and oil emulsions. This phenomenon has contributed to increased interest in the processes of microorganism development in petroleum products^12,13^.

Microorganisms are present in all types of natural environments, such as water, soil and air, from where they can enter crude oil and its products at various stages of production, distribution, storage and use. Oil used in engines is additionally exposed to high temperatures and combustion products, and may also be diluted by fuel. An additional risk is posed by adverse interactions between oil and fuel entering the lubrication system, which can lead to accelerated degradation of lubricants. For this reason, high-quality fuel should be clear and free of cloudiness. The presence of discolouration or cloudiness most often indicates the growth of microorganisms, which can then penetrate the engine oil and intensify biological degradation processes in it^14,15^. Additional difficulties in this area are associated with the growing use of biofuels. Due to varying degrees of oxidation, resulting from both their accelerated ageing and incomplete combustion in engine chambers, they can penetrate lubricating oil, accelerating its degradation process. The problem of microbiological contamination of petroleum products will increase as the use of alternative fuels, including biofuels, becomes more widespread for environmental reasons. Compared to diesel fuel, biodiesel and its blends are more susceptible to microbial degradation^16–18^. This is because biofuels are a much better source of carbon for microorganisms than traditional diesel fuel. The activity of microorganisms leads to the degradation of both hydrocarbons and additives, as well as the release of water, sulphur compounds and surfactants into petroleum products. These processes cause changes in the chemical composition of fuels and oils, while also affecting their key physical properties, such as flash point, base number and viscosity^19,20^.

Since the quality of the diesel fuel used has a significant impact on the properties of the lubricating oil, this translates into difficulties in maintaining its performance parameters at the required level. Currently, biocides – chemicals classified as pesticides – are used to limit the growth of microorganisms in petroleum products. However, it should be emphasised that most of them also destroy beneficial microorganisms and can lead to adverse changes in the structure of microflora. For this reason, despite the benefits of their use, the scope of application of biocides is limited, mainly due to concerns about their negative impact on the environment. As a result, the search for alternative methods of combating microbial contamination that are environmentally safe and do not eliminate beneficial microorganisms is becoming increasingly important^21–23^.

In summary, it is important to emphasise the need to develop effective methods to counteract microbial contamination in petroleum products. These solutions should take into account both technical aspects and the specific nature of the processes occurring in lubricating oils and lubrication systems, while remaining safe for the environment^24,25^.

Taking the above conditions into account, research was conducted on the impact of additives in the form of ionic and non-ionic silver and effective microorganisms, both in liquid form and in the form of ceramic tubes, after the oil had been used in a combustion engine. Their impact on selected oil parameters and their effectiveness in limiting the growth of bacteria and fungi were analysed. Both microorganisms and silver compounds are used in many other areas and are widely recognised as environmentally friendly solutions.

Both silver compounds and effective microorganisms (EM) have previously been studied in the context of environmental protection, agriculture and medicine, but their use as ecological additives to lubricating oils has not yet been analysed. This paper presents the first results of research on the effect of silver compounds (ionic and non-ionic) and EM in liquid and ceramic form on the physicochemical and microbiological stability of engine oil used in marine engines. This interdisciplinary approach, combining tribology, microbiology and environmental engineering, is an innovative alternative to traditional chemical biocides, and the use of EM in ceramic form is an original solution, not yet described in the scientific literature. The proposed approach combines environmentally friendly biocidal mechanisms with tribological assessment, providing an innovative perspective on lubricant engineering.

## Materials used in the research

**MARINOL RG 1230** is a marine engine oil intended for lubrication of marine stern engines running on light fuel. This oil contains a suitably selected set of detergent and dispersant, antioxidant, anti-corrosion, anti-rust and anti-wear additives. Marinol RG 1230 is a trunk piston engine oil that meets the requirements of API CF for marine oils^26^. Typical oil parameters are given in Table 1.

The technology applied in **MARINOL RG 1230** oil provides the following benefits:

- Effective lubrication of the engine cylinder system,
- High engine cleanliness combined with reliable corrosion protection,
- Reduction of deposit and carbon build-up contributing to engine wear,
- Minimization of both low- and high-temperature deposits formed during engine operation,
- Neutralization of acidic combustion by-products,
- Decreased frequency of oil top-ups.

**Table 1:** Parameters of the tested engine oil^26^.

Parameters	Test methods	Unit	1230
Kinematic viscosity at 100 °C	ASTM D 445	mm^2^/s	11.5
Pour point	ASTM D 5950	^o^C	-24
Flash-point	PN-EN ISO 2592	^o^C	240
Base number TBN	PN-ISO 3771	mg KOH/g	12
Viscosity index	ASTM D 2270		99

One of the additives introduced to the lubricating oil was effective microorganisms (EM) – a complex of naturally occurring, non-genetically modified cultures of beneficial microorganisms. These organisms are safe for humans, animals and the environment, and their presence is essential for the proper functioning of biological processes. EM have been carefully selected and are used in horticulture, environmental protection, medicine, industry and other sectors of the economy. Photosynthetic bacteria play a special role in the mixture, transforming organic matter and toxic gases into biologically active, beneficial compounds under the right conditions (CO₂, temperature). Lactic acid bacteria, which inhibit the growth of pathogenic microorganisms, and yeasts, which support the decomposition of organic matter and neutralise unwanted odours through fermentation processes, are also important components. The principle of action of Effective Microorganisms (EM) is based on natural biological processes that do not interfere with the genotype of organisms and are completely safe for the environment. EM preparations are mainly used in water treatment, sewage treatment and the restoration of water reservoirs. This technology is also used in waste incineration plants, where it is highly effective in reducing toxic dioxin emissions. The literature also emphasises the importance of heavy metals such as copper and silver in combating bacteria, fungi and viruses^27,28^. The study used a preparation with the following composition: water – 94%, Effective Microorganisms^®^ – 3%, molasses – 3%.

Effective microorganisms were used in two forms: liquid (250 ml per 15 L of oil) and ceramic tubes (500 g, 9 mm in diameter and 11 mm in height per 15 L of oil). The additives were introduced into fresh oil, which constituted approximately 2% of the total oil volume in the engine. The number of ceramic tubes was greater than the amount of additive in liquid form, due to the fact that microorganisms embedded in the ceramic structure are released into the oil in a different way than in the case of the liquid preparation.

**Fig. 1:**
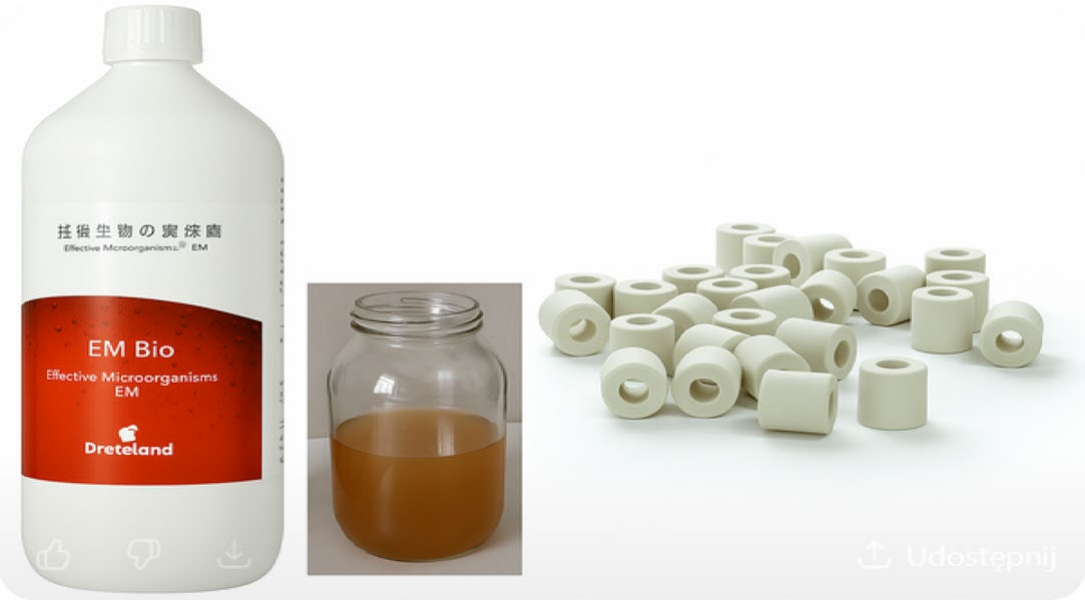
The commercial form of effective microorganisms in liquid form and ceramic tubes^29,30^.

For the purpose of the study, **effective microorganisms in their commercial form** (shown in Fig. 1) were applied.

The second additive introduced to the fuel was silver, an element with a long tradition of use for protective and medicinal purposes. It is used, among other things, in filters and for disinfecting air and water in enclosed spaces, such as aeroplanes. The ionisation process further enhances its biocidal properties. As with copper, ionised forms of silver have been used to eliminate specific types of bacteria. Research has shown that copper and silver ions can penetrate biofilms in water supply systems, effectively limiting the growth of microorganisms. Experiments conducted on hospital water enriched with these elements confirmed a reduction in the number of bacteria and fungi by approximately 30% compared to samples in which they were not used^31^.

The study used silver in two forms: solution and colloidal nanosilver. Colloidal silver (also known as nanosilver or collargol) is an aqueous colloid of silver nanoparticles with a diameter of 5–15 nm. These particles are non-ionic, remaining suspended in demineralised water without forming permanent chemical bonds with its molecules. Due to their equal electrical charge, the nanoparticles repel each other, which keeps them in constant motion and prevents them from agglomerating. Non-ionic colloidal silver is obtained by electrolytically dissolving a silver electrode in water, which leads to the formation of stable nanoparticles that do not exhibit chemical reactivity and are considered safe for the body. A characteristic feature of this solution is its dark yellow colour, resulting from the scattering of light with a wavelength of approximately 400 nm by the silver particles present in it.

**Fig. 2:**
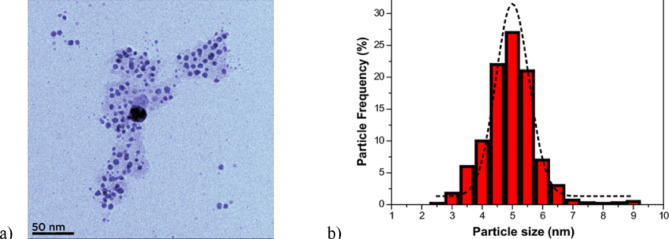
Transmission electron microscope (TEM) image of silver nanoparticles (**a**) and particle size distribution (**b**) of Ag-NPs synthesized under optimal conditions^32^.

Figure 2 presents a transmission electron microscopy (TEM) image of silver nanoparticles along with their size distribution^32^.

The size distribution of silver nanoparticles shown above was performed using an ultrasonic synthesis technique using honey as a reducing and encapsulating agent. These results can be extended to other precious metals such as gold, palladium and copper, offering various additional applications from medicine to industry.

The literature distinguishes between two basic forms of silver preparations used for biocidal purposes: colloidal nanosilver and silver ion solution^33–35^:

- **Colloidal nanosilver** consists of approximately 80% silver nanoparticles and 20% silver ions. Its quality depends on the number of particles and the total active surface area available for reaction. In this type of preparation, silver particles form a colloid without the participation of a protein stabilising substrate. Due to their size (from several dozen to several thousand times smaller than bacteria and viruses), silver nanoparticles can penetrate microorganisms, damaging and eliminating them,
- **Silver ion solution** has different properties: it is as clear as water, and its composition includes approx. 90% silver ions and only 10% silver particles. Due to the predominance of ions, it would be more appropriate to refer to it as a ‘silver solution’. It is formed as a result of chemical processes in which a silver atom loses an electron, giving it the ability to enter into chemical reactions.

**Fig. 3:**
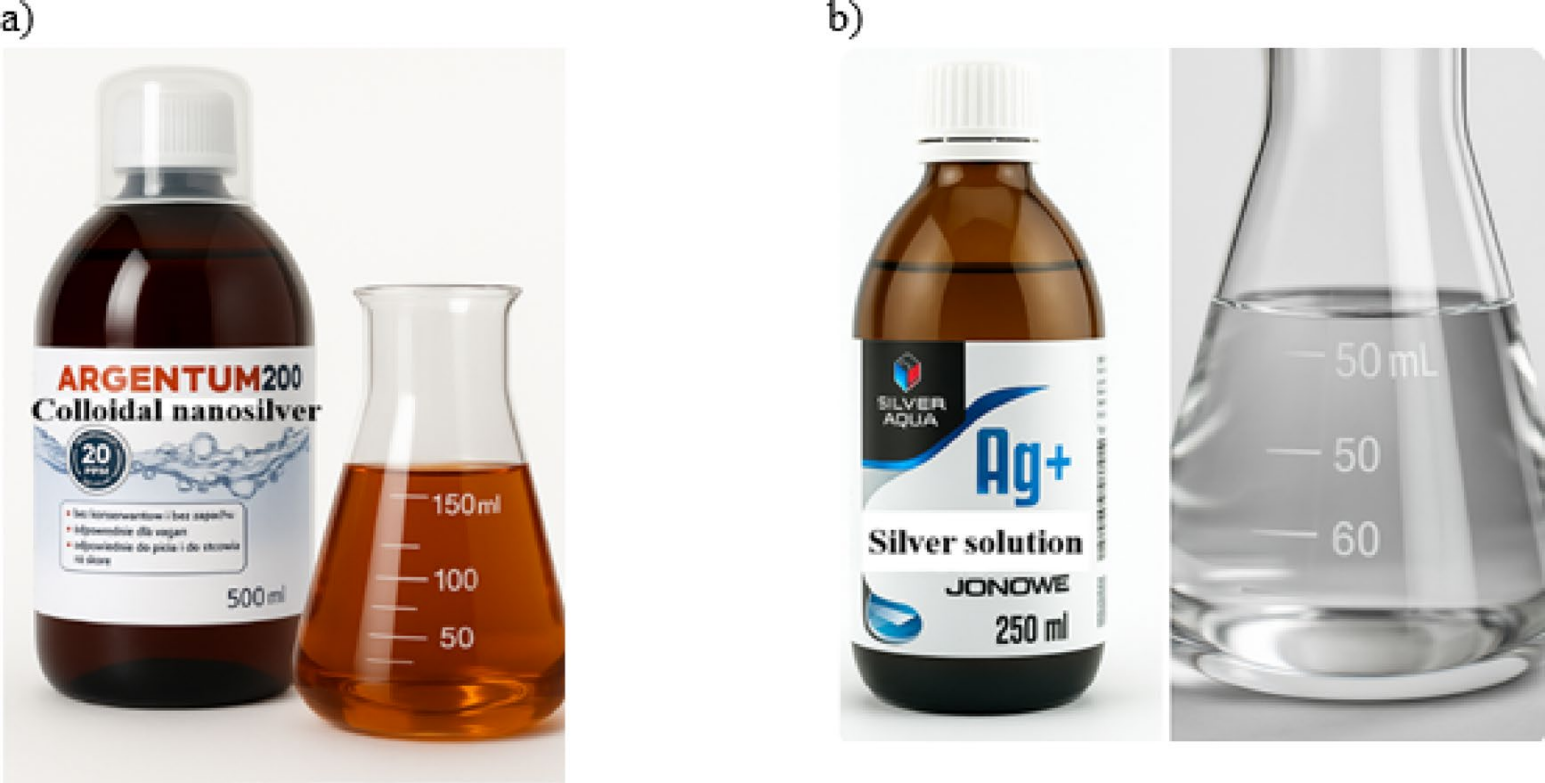
The commercial form of colloidal nanosilver and silver solution^36,37^.

Silver was added to fresh oil in amounts corresponding to the doses of effective microorganisms. The study used a commercially available silver preparation, shown in Fig. 3.

.

To date, there is no literature data on the use of the described additives and their impact on the properties of lubricating oils and the rate of microorganism growth. The introduction of these substances can therefore be considered an innovative solution that could potentially contribute to reducing the activity of undesirable bacteria and fungi in engine oil.

In the future, in-depth research will be necessary to assess their impact on the physicochemical stability of oil, tribological properties, service life and long-term effectiveness in reducing microbial contamination.

### Mechanism of action of ecological additives

The observed antimicrobial properties of the tested additives result from different but complementary mechanisms of action. Silver-based additives work mainly by releasing Ag⁺ ions, which penetrate the cell membranes of microorganisms and bind to the thiol groups of proteins, enzymes and DNA. This causes damage to cell membranes, inhibition of enzymatic activity and replication. In addition, the resulting reactive oxygen species (ROS) increase oxidative stress within the cells, leading to their deactivation. Colloidal silver, thanks to its large specific surface area, ensures controlled and long-lasting release of ions, maintaining biocidal activity over time.

Effective microorganisms (EM) work biologically – on the basis of metabolic competition and environmental regulation. A consortium of lactic acid bacteria, photosynthetic bacteria and yeast produces organic acids, enzymes and antioxidants that limit the growth of undesirable microflora and stabilise the oil environment. When introduced into lubricating oil, EMs limit the growth of bacteria and fungi by modifying the redox potential and reducing the formation of organic deposits.

The simultaneous use of silver compounds and EM can produce a synergistic effect – rapid biocidal action combined with long-term oil stabilisation, providing an environmentally friendly alternative to traditional chemical biocides. To gain a more comprehensive understanding of the mechanisms of action of these additives, further research into their chemical and physical effects on engine oil is recommended. Therefore, additional studies are planned on the practical application of engine oils containing these additives in order to verify the presented results under actual operating conditions. In addition, it is necessary to investigate the mechanisms of action of the additives, i.e. the effect of combining two or three additives that have shown the most beneficial effect on the tested properties of engine oil. It is also important to investigate the effect of these additives on the technical condition of the internal combustion engine and the wear of its components.

### Technical and economic analysis

From a technical point of view, both additives tested – silver compounds and effective microorganisms – can be used in existing lubrication systems without the need to modify the engine design. Their application is simple and can be carried out during routine oil changes or regeneration.

The estimated cost of silver- and EM-based additives at a concentration of 2% currently accounts for approximately 3% of the total oil input value. However, it should be emphasised that the additives were purchased in retail quantities for laboratory testing purposes. In the case of industrial implementation, oil producers would purchase them in bulk quantities, which would significantly reduce the unit cost – probably to less than 3% of the total input cost.

In addition, extending oil life and improving its microbiological stability can reduce replacement frequency and maintenance costs by 5–10%, which partially offsets the cost of additives. The use of EM ceramics, which can be reused multiple times, further increases the cost-effectiveness of the solution. From an environmental point of view, replacing traditional chemical biocides with biodegradable additives with low toxicity is in line with the principles of sustainable development and reduces the environmental footprint of vessel operation.

### The research stand and methodology

Before starting the tests, tanks containing engine oil were prepared, into which four types of additives were introduced: effective microorganisms in liquid form, ceramic tubes, silver in the form of an ionic solution, and colloidal nanosilver (non-ionic silver). These substances were dosed at a concentration of 2% into tanks with a capacity of 20 dm³. This concentration was chosen because these additives had not previously been used in petroleum products, and therefore their effect on the properties of lubricating oil remains unknown. Additionally, it should be emphasised that increasing the percentage would not only entail the risk of undesirable effects on the oil, but also a significant increase in costs, which could limit the profitability of their practical application.

To maintain a uniform consistency, the prepared mixtures were homogenised twice a day for 15 min at 20 ± 2 °C using a POLYTRON PT 45–80 GT laboratory homogeniser. The samples were stored under controlled conditions: temperature 20 ± 2 °C, relative humidity 40–60%, dark room, no UV light. The exposure time before testing was 4 weeks. Just before testing, the mixtures intended for analysis showed no signs of instability. Four test samples were prepared for the experiment: engine oil with the addition of effective microorganisms in ceramic and liquid form, as well as oil with the addition of silver solution and colloidal nanosilver.

For better clarity, the following codes have been used in the article for each sample, which are shown in Table 2.

**Table 2:** List of oil samples tested with their assigned codes.

Sample description	Sample code
New engine oil without additives	NO
New engine oil + silver solution	NO + SS
New engine oil + colloidal nanosilver	NO + CN
New engine oil + effective liquid microorganisms	NO + EM(F)
New engine oil + effective microorganisms in ceramics	NO + EM(C)
Used engine oil without additives –	UO
Used engine oil + silver solution	UO + SS
Used engine oil + colloidal nanosilver	UO + CN
Used engine oil + effective liquid microorganisms	UO + EM(F)
Used engine oil + effective microorganisms in ceramics	UO + EM(C)

**Fig. 4:**
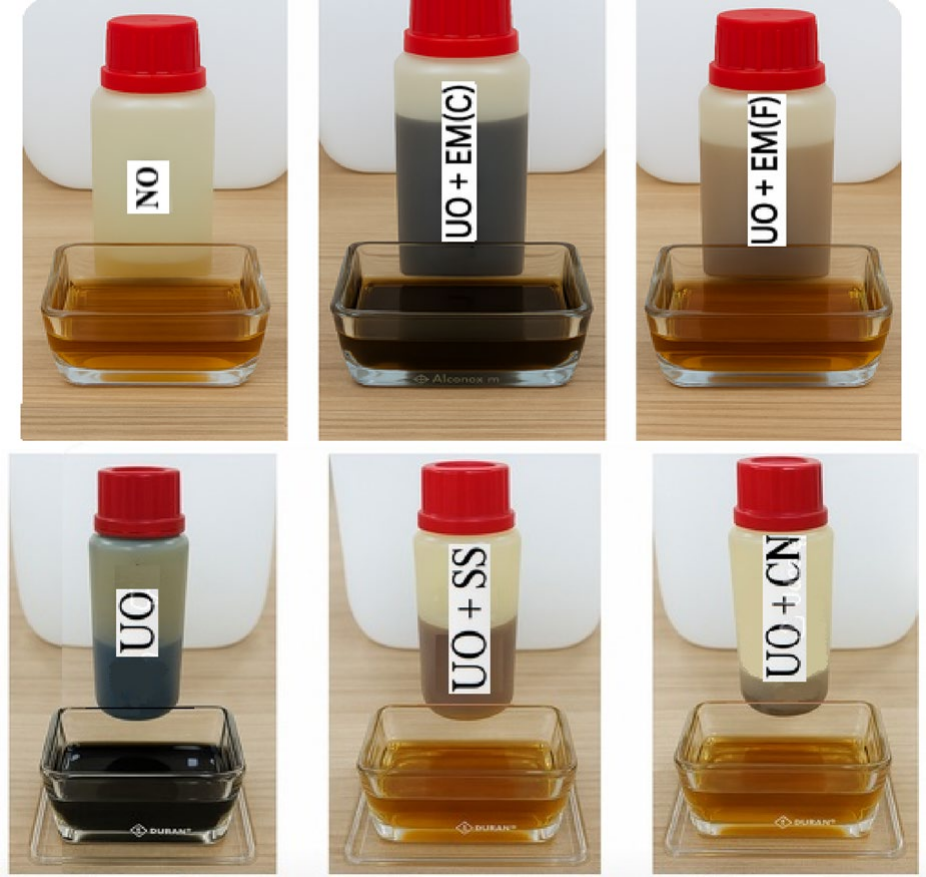
Pure oil, oil with the addition of effective microorganisms (EM) and silver (before and after working), where SS – silver solution, CN – colloidal nanosilver.

Figure 4 presents samples of fresh oil, fresh oil with additives (before being introduced into the engine), and used oil (after engine operation). It was observed that the colour and appearance of the used oil remained unchanged, regardless of the type of additive applied.

The article presents the results of tests, which began with a control test. The following oil parameters were assessed: flash point, water content, acid and base number, as well as density and kinematic viscosity. Pure lubricating oil, free of any additives, was used for the control test. Physicochemical analyses were carried out in the laboratories of the Gdynia Maritime University, while microbiological tests, due to the lack of appropriate equipment, were commissioned to a specialised external company^38^.

The tests were conducted under controlled environmental conditions: the operating temperature of the engine oil was 77 °C during idling and approximately 79 °C under a load of 20 kW, while the coolant temperature remained at 76 °C during idling and 77 °C under load. The ambient temperature throughout the experiment was 20 °C.

Before commencing the actual testing phase, each oil sample — starting with the pure oil sample, followed by oil samples with individual additives — was tested in an engine under identical operating conditions. Each test cycle consisted of 100 h of engine operation (excluding breaks), including 80 h of idling and 20 h at 50% of the nominal load (20 kW), maintained at a constant level throughout the test. The engine speed was constant at 1500 rpm.

After the planned operating time had elapsed, the oil was drained from the engine and subjected to laboratory analysis to assess the effect of the additives used on selected physicochemical parameters. The results obtained were compared with the values obtained for oil without additives.

The SW 680/1 Delfin, a low-speed direct injection diesel engine manufactured by WSK Mielec under licence from British Leyland, was used for the tests. This unit is a six-cylinder in-line engine with a rated power of 147 kW at 2,200 rpm. The engine was mechanically coupled to a three-phase synchronous generator GCPf94c/1 with a rated power of 60 kVA, which was electrically connected to the main switchboard. The engine load was smoothly regulated by changing the electrical load of the generator using an adjustable resistor. The fuel supply system included a piston injection pump and CAV injectors, manufactured under licence from Friedmann-Meyer^39^.

**Fig. 5:**
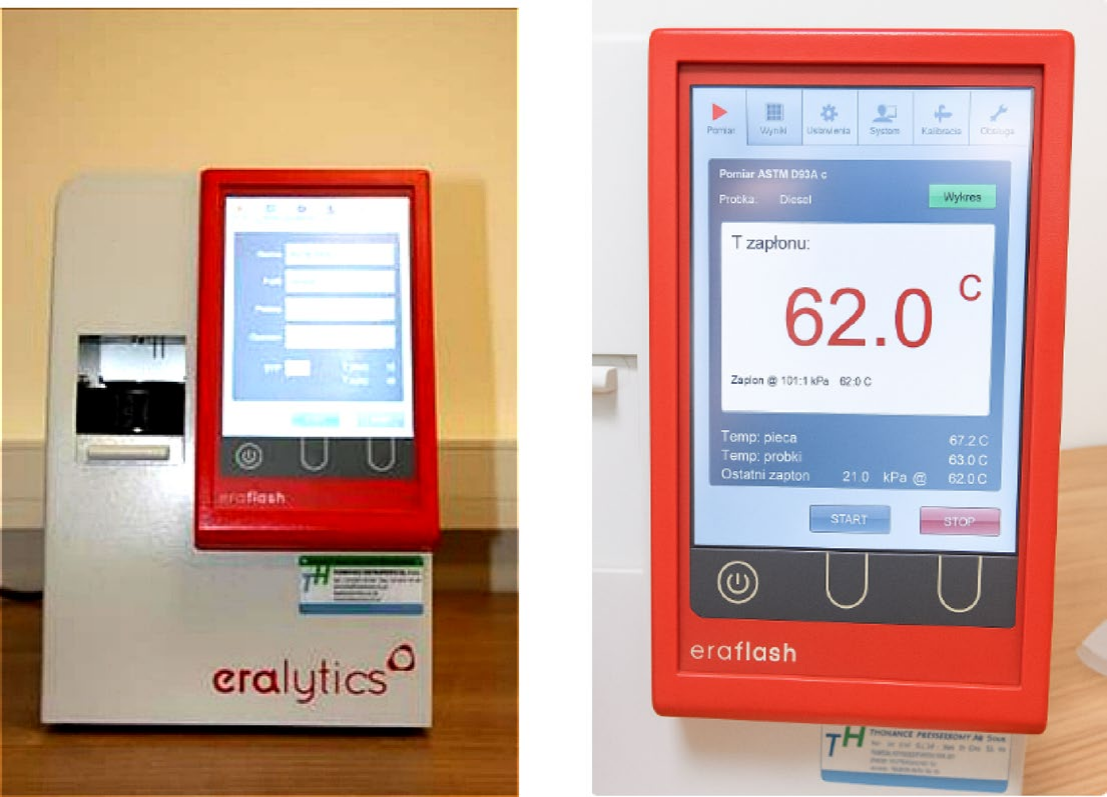
Apparatus for testing the flash point in a closed cup – EraFLASH^39,41^.

The flash point^40^ of pure engine oil and oil with additives was determined using an automatic apparatus with a closed crucible (Fig. 5). The device used is characterised by high precision and measurement speed, patented heating and cooling technology in a wide temperature range from − 25 °C to + 420 °C (in a single device), as well as the ability to implement methods compliant with ASTM D 6450 and ASTM D 7094 standards.

The device is designed to determine the flash point of various petroleum products. It is easy to use and can also be used in field conditions. A small sample volume is required (1 ml according to ASTM D 6450 or 2 ml according to ASTM D 7094), and the use of a closed crucible during measurement significantly increases the level of laboratory safety. The sample is heated from above in a sealed measuring chamber.

Ignition is initiated by an electric arc, while the ignition temperature is defined as the point at which a rapid increase in gas pressure is observed in the measuring chamber. The tests on the samples used to prepare the graphs and analyses were carried out in accordance with the requirements of ASTM D7094.

Engine oil always contains a certain amount of water, the concentration of which should be kept at a safe level, well below the saturation point. The Karl Fischer titration method, used in laboratories since 1935, is commonly used for the precise determination of water content in liquid samples. This method is highly sensitive and accurate, requires a small sample volume and allows the detection of even trace amounts of dissolved water — down to a level of approximately 50 ppm in diesel fuel. It enables the detection of both dissolved water (below the saturation point) and free water (above the saturation point). It is crucial to maintain the water content below the saturation point, which allows it to remain in a dissolved state and prevents free water from entering the lubrication systems.

**Fig. 6:**
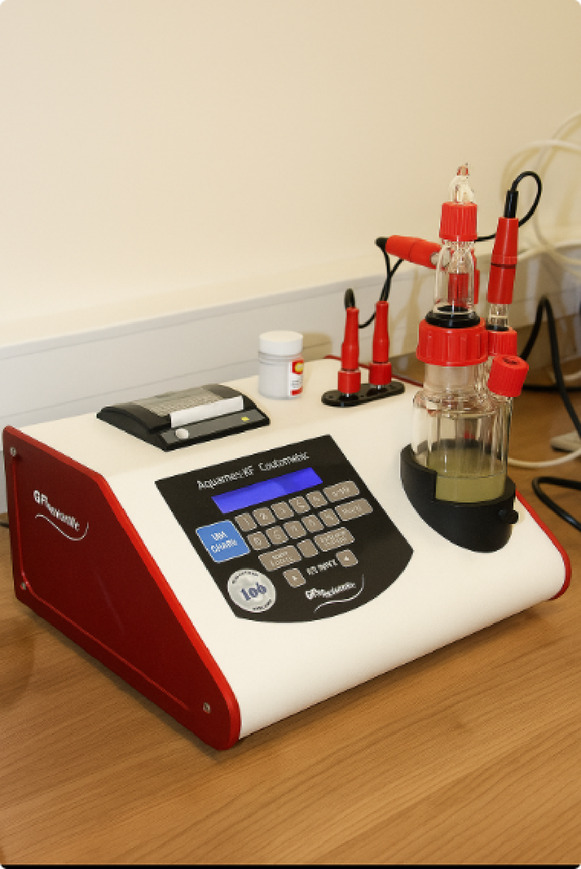
Tested oil with solvent and iodine on automatic Cou-Lo AquaMax^42^.

The water content in diesel fuel, both with and without additives, was determined using an automatic Cou-Lo AquaMax KF titrator, operating on the basis of Karl Fischer’s method^42^. This device (Fig. 6) uses coulometric titration, which allows for precise and reliable results in the range from 1 ppm upwards. The measurement takes place in a sealed vessel in which iodine is produced electrolytically, which minimises the influence of ambient moisture (so-called ‘drift’). Selected technical parameters of the device are presented in Table 3.

**Table 3:** Selected parameters of the Cou-Lo AquaMax KF automatic titrator^42^.

Parameters	Cou-Lo aquamax KF moisture meter
Titration method	Coulometric Karl Fischer titration
Endpoint detection	AC polarity
Measurement range	1 µg – 10 mg of water, max 200 mg of water
Moisture range	1 ppm – 100% water
Maximum titration rate	2.24 mg per minute
Maximum electrolysis current	400 mA
Drift Compensation	Controlled automatically
Minimum titration time	0–30 min, user programmable
Precision	10–100 µg ± 3 µg, 100 µg–1 mg ± 3 µg (ppm), over 1 mg ± 0.3%
Display format	µg, mg/kg, ppm, %

The measuring system uses a platinum electrode that generates an electrical charge and precisely releases stoichiometric amounts of iodine into the ceramic membrane system. This process is based on the relationship whereby a charge of 10.71 C corresponds to an amount of iodine equivalent to 1 mg of water. The AquaMAX KF apparatus operates on the principle of coulometry, determining the water content in a sample by coulometric titration until the end point is reached^42^.

**Fig. 7:**
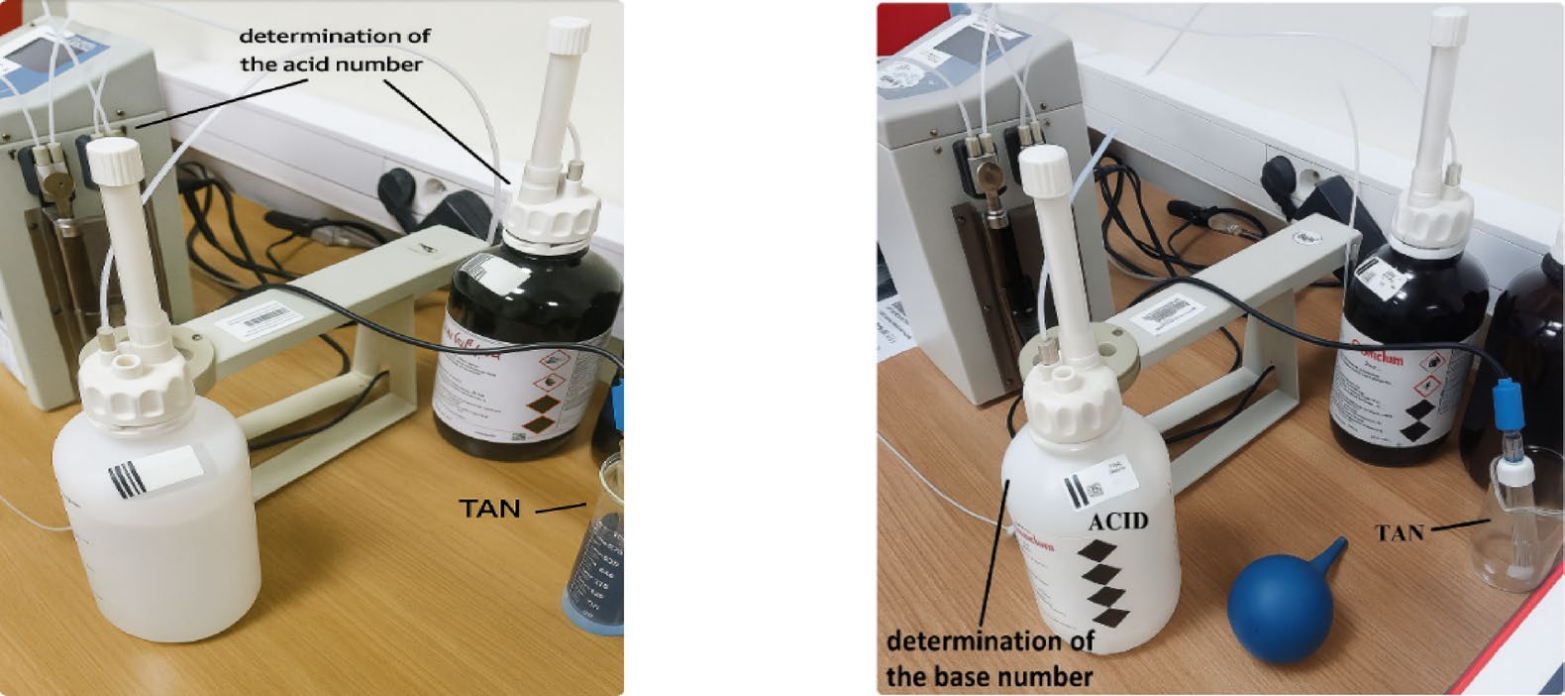
Titrator TitroMatic 2 S device prepared to determine the acid number.

The research also determined the acid and base numbers of the engine oil using a TitroMatic 2 S titrator (Fig. 7).

The TitroMatic 2 S automatic titrator is equipped with two interchangeable piston burettes that can operate both independently and simultaneously. In addition, the device has a peristaltic pump for automatic reagent dosing, analysis support and precise determination of the sample volume before titration. The titrator automatically feeds the titrant into the system until the predetermined titration endpoint is reached, defined by the pH value (e.g. 8.10 pH for acidity determination) or mV potential (e.g. 150 mV for chlorides and SO₂)^43^.

Before starting the tests, the titrator was started up, the titrant (potassium hydroxide) and the measuring electrode were connected, and then the device was calibrated using standard solutions with known pH values. After completing these steps, the actual titration could begin. First, a blind test was carried out in a T.A.N. mixture. Then, using a syringe, 20 ml of the test oil was collected, placed in a pre-weighed measuring cup, and the empty syringe was weighed. After a few minutes of titration, the apparatus indicated the acid or base number, depending on the measurement configuration and appropriate sample preparation.

Microbiological testing of lubricating oil was performed in accordance with the standard procedure for determining live bacteria and fungi in petroleum products, based on the membrane filtration and culture method specified in ASTM 6974-3. This procedure uses a membrane filter (MF) to detect and determine the number of heterotrophic bacteria (HPC) and fungi in liquid fuels with a kinematic viscosity ≤ 24 mm²∙s⁻¹ at ambient temperature. The ability of microorganisms to form colonies on specific media depends on their taxonomy and physiological state, as well as on the chemical composition of the medium and incubation conditions. For this reason, test results should be treated with caution – not as absolute values, but as part of diagnostics and monitoring, in conjunction with other test parameters, in accordance with the guidelines of D 6469.

As part of microbiological testing, the number of aerobic microorganisms, including both bacteria and fungi, was determined in fuel samples incubated at 25 °C. The analyses were performed in two volume variants, with one repetition for each volume, using an optimisation procedure. The optimisation consisted of a two-stage determination: initially using a larger sample volume, and then, after obtaining the results, using a smaller volume. In stages II and III, 1 ml of the sample was filtered, with additional dilutions performed. The equipment set included a class II laminar flow cabinet and a membrane filtration kit. All samples were subjected to aseptic mixing before analysis. In stage I, after homogenisation, five samples, each with a volume of 10 ml, were filtered (due to the lower density of the material).

In stage II, due to the nature of the samples (density), membrane filtration was used as part of optimisation aimed at obtaining more accurate results, preceded by the classic procedure of tenfold dilutions. Membranes with a pore diameter of 0.45 μm were used for the analysis, which were washed with Tween 20 cleaning fluid and then with Ringer’s solution before use. The samples were filtered repeatedly (4 ml and 5 ml onto separate filters, respectively) and then incubated at 25 °C. Readings were taken after 72 h and after another 5 days. The results were presented as the number of bacteria or fungi in 1 dm³ of fuel, assuming that each colony grew from a single cell. The membrane filters were placed on appropriate culture media: TSA (tryptone soy agar) for bacteria and MEA agar (or its alternative) for fungi, which ensured optimal growth conditions for both groups of microorganisms. In stage III, the tenfold dilution method was again used with Tween 20 solution. The final results were presented as average values from stages I–III, taking into account only quantifiable values^38^.

In order to assess the impact of the additives used, a statistical comparison of engine oil samples was performed, assuming a significance level of α = 0.01. The analysis of differences in oil properties before and after the addition of additives was performed using a paired Student’s t-test. The null hypothesis (H₀) assumed that there were no significant differences between the mean values of the tested groups. If the obtained p-value was greater than the accepted significance level (*p* > 0.01), it was concluded that the additives had no statistically significant effect on the properties of the oil. However, when the p-value was less than the significance level (*p* ≤ 0.01), it was concluded that there were grounds for rejecting the null hypothesis and that the additives had a significant negative effect on the functional properties of the oil.

## Results and discussion

The article presents the results of tests of selected parameters of pure lubricating oil in comparison with oil modified with ecological additives (effective microorganisms, colloidal nanosilver). The following parameters were assessed: flash point, water content, acid and base number, density and kinematic viscosity. In addition, diesel fuel was tested for microbial degradation.

### Microbiological tests

Engine oil samples were subjected to microbiological testing to determine the presence of live bacteria and fungi in accordance with ASTM D 6974-3. After filtering a specified volume of samples through a membrane filter, the microorganisms were placed on culture media, providing them with suitable conditions for growth. The results obtained are summarised in Table 4.

**Table 4:** Results of microbiological tests.

Sample description	Bacteria (B)/fungi (F)	Microbial count results in temp. 25±2 °C [ cfu/dm³ ]
NO	B	1.6 × 10^6^
	F	1.8 × 10^4^
NO + SS	B	1.5 × 10^6^
	F	1.1 × 10^3^
NO + CN	B	5.3 × 10^5^
	F	6.7 × 10^3^
NO + EM(F)	B	1.6 × 10^6^
	F	5.9 × 10^3^
NO + EM(C)	B	5.3 × 10^5^
	F	4.6 × 10^3^
UO	B	1.5 × 10^7^
	F	3.6 × 10^5^
UO + SS	B	3.8 × 10^5^
	F	3.1 × 10^4^
UO + CN	B	2.5 × 10^6^
	F	1.6 × 10^4^
UO + EM(F)	B	1.7 × 10^4^
	F	7.7 × 10^3^
UO + EM(C)	B	2.5 × 10^6^
	F	5.5 × 10^3^

Microbiological analyses demonstrated that all tested additives contributed to reducing the microbial contamination of the engine oil compared with the untreated control. For fresh base oil without additives, the bacterial count was 1.6 × 10⁶ CFU/dm³, and the fungal count was 1.8 × 10⁴ CFU/dm³. After 4 weeks of operation without additives, microbial contamination increased markedly − bacterial counts rose approximately ninefold to 1.5 × 10⁷ CFU/dm³, while fungal counts increased about twentyfold to 3.6 × 10⁵ CFU/dm³. The addition of liquid effective microorganisms (EM) did not significantly affect the bacterial population but reduced fungal growth approximately threefold, to 5.9 × 10³ CFU/dm³. The application of ceramic EM resulted in a threefold reduction in bacterial counts (5.3 × 10⁵ CFU/dm³) and a fourfold decrease in fungal counts (4.6 × 10³ CFU/dm³) relative to the control sample. The use of the silver solution slightly lowered the number of bacteria (1.5 × 10⁶ CFU/dm³, ~ 10% decrease) but led to a sixteenfold reduction in fungi (1.1 × 10³ CFU/dm³). A comparable trend was observed for colloidal nanosilver, which reduced bacterial contamination roughly threefold (5.3 × 10⁵ CFU/dm³) and fungal counts about threefold (6.7 × 10³ CFU/dm³). These results indicate that while all tested additives inhibited microbial growth to some extent, the silver-based formulations exhibited the strongest antifungal activity, and ceramic EM provided a balanced antibacterial and antifungal effect.

The application of all tested additives resulted in a measurable reduction in the number of bacteria and fungi compared with the oil used without additives. Among them, the silver solution exhibited the most pronounced antibacterial effect, reducing the bacterial count approximately fortyfold to 3.8 × 10⁵ CFU/dm³ and the fungal count about twelvefold to 3.1 × 10⁴ CFU/dm³. The use of colloidal nanosilver and ceramic effective microorganisms (EM) led to similar sixfold decreases in bacterial counts, reaching 2.5 × 10⁶ CFU/dm³. However, their antifungal efficiency differed: colloidal nanosilver reduced the fungal population approximately twenty-twofold (1.6 × 10⁴ CFU/dm³), whereas ceramic EM achieved a sixty-fivefold reduction (5.5 × 10³ CFU/dm³), indicating strong inhibitory potential against fungal growth. The addition of liquid EM also produced a clear antimicrobial effect, with bacterial counts decreasing ninefold (1.7 × 10⁶ CFU/dm³) and fungal counts decreasing forty-sevenfold (7.7 × 10³ CFU/dm³) compared with the oil without additives. These results confirm that all tested additives contributed to lowering microbial contamination of the engine oil, though to varying degrees depending on the additive type. The post-operation analyses are particularly significant, as they reflect the actual behavior of the additives under conditions that promote oil degradation. In this regard, ionic silver appeared to provide the most effective bactericidal protection, whereas ceramic EM showed superior antifungal performance. Consequently, oil samples containing these additives exhibited the smallest deviations in physicochemical parameters and the lowest accumulation of microbial residues (sludge).

### Flash point of lubricating oil

**Fig. 8:**
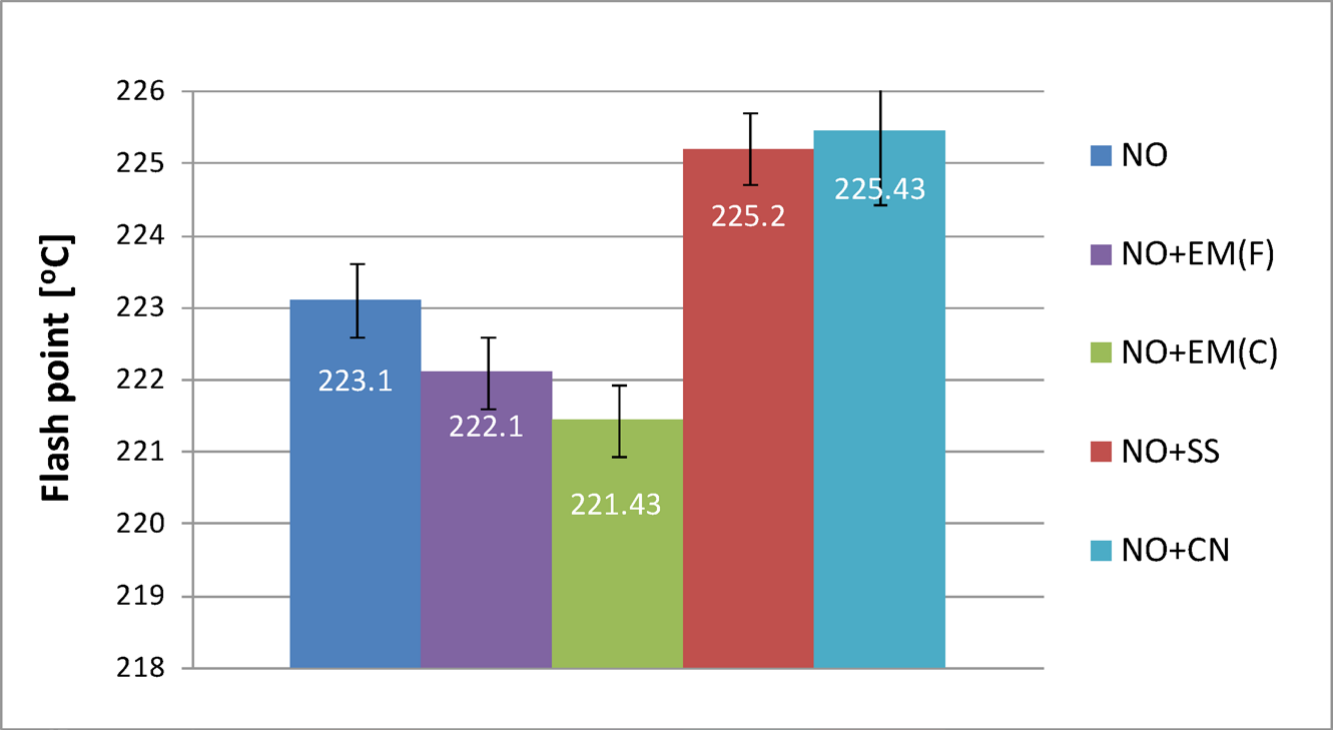
Flash point of pure new engine oil and engine oil with additives in a concentration of 2%.

To evaluate the flash point of the lubricating oil in the tested samples, measurements were carried out at a 2% concentration of each additive. The results, presented in Fig. 8, show variations in ignition temperature for the base oil and oils supplemented with effective microorganisms (EM) in liquid and ceramic forms, as well as with silver solution and colloidal nanosilver. Each variant − including the base oil − was tested in triplicate, and the average values are reported. The flash point of the pure oil was 223.1 °C. The addition of liquid EM at 2% of the total oil volume (15 dm³) slightly lowered the flash point to 222.1 °C, representing a decrease of approximately 1.0 °C (0.45%) compared with the base oil. The use of ceramic EM resulted in a further reduction to 221.4 °C, the lowest value among all analyzed samples (a decrease of 1.7 °C, or 0.76%, relative to the control). In contrast, oils enriched with silver solution and colloidal nanosilver showed slightly higher ignition temperatures compared with the base oil, indicating that these additives did not negatively affect the thermal stability of the lubricant.

The addition of the silver solution increased the flash point to 225.2 °C, representing a rise of 2.1 °C (0.94%) compared with the base oil. The highest ignition temperature was recorded for the sample containing colloidal nanosilver, which reached 225.4 °C, i.e., 2.3 °C (1.03%) higher than that of pure oil. The flash point value closest to that of the base oil was obtained for the sample containing liquid EM (222.1 °C). Although this temperature differed by only 1.0 °C from the reference, the observed reduction indicates a slightly lower resistance of the oil to high-temperature exposure. Therefore, this type of additive may require careful consideration for use in internal combustion engines, where a reduced flash point could potentially increase the risk of vapor ignition under extreme conditions.

Furthermore, Table 5 presents the flash point results for new oil together with basic statistical analysis.

**Table 5:** Flash point results for new oil with basic statistical analysis.

	Flash point of new oil		
	Value average	Standard deviation	*P*-value
NO	223.10	1.76	–
NO + EM(F)	222.10	1.00	0.98
NO + EM(C)	221.43	0.57	0.55
NO + SS	225.43	1.00	0.06
NO + CN	225.20	1.15	0.05

The data presented in Table 5 indicate that both forms of effective microorganisms (EM) − liquid and ceramic − lowered the flash point compared with the base oil. This decrease may be associated with a slight reduction in thermal stability, which could be disadvantageous for high-temperature engine applications. In contrast, silver-based additives, i.e., silver solution (SS) and colloidal nanosilver (CN), produced the opposite effect by increasing the flash point, suggesting improved resistance of the oil to ignition at elevated temperatures. The observed increase was practically meaningful and approached statistical significance (*p* ≈ 0.05).

**Fig. 9:**
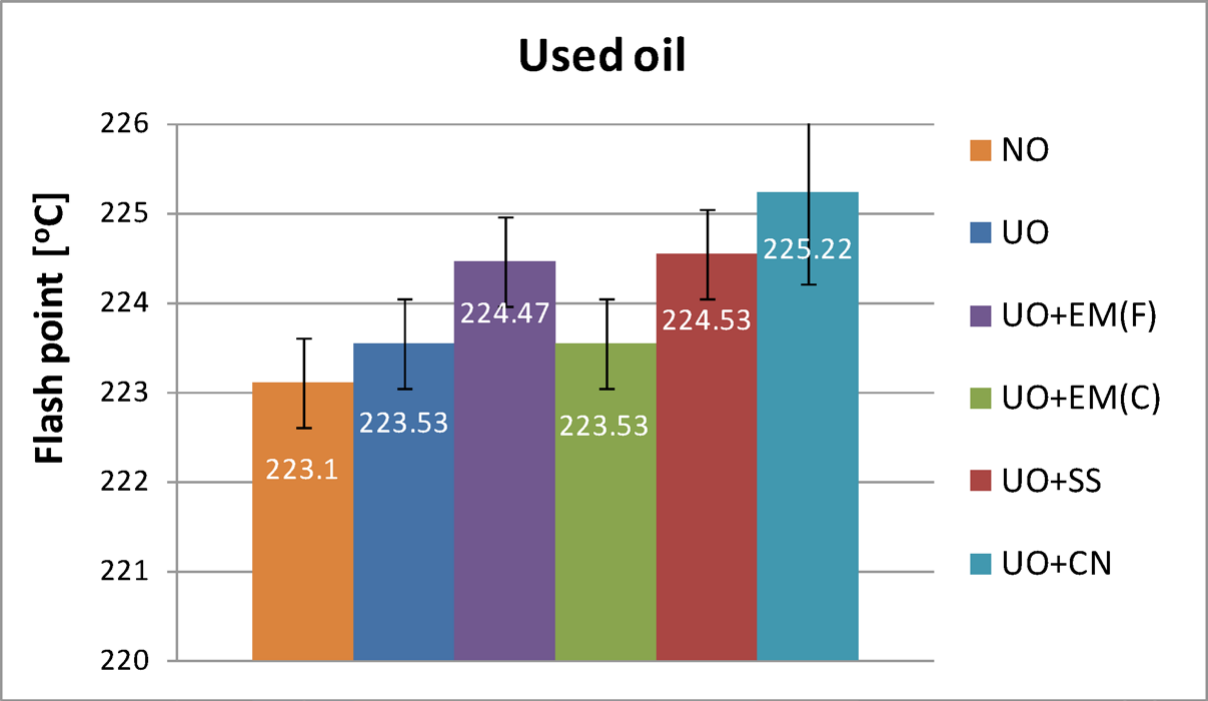
Flash point of pure used engine oil and engine oil with additives in a concentration of 2%.

Figure 9 shows the flash point values for used lubricating oils, both pure and with additives, each at a 2% concentration. Oils containing liquid EM and ionic silver exhibited nearly identical flash point values (224.47 °C and 224.53 °C, respectively), approximately 1.0 °C higher than the used oil without additives (223.5 °C). The addition of ceramic EM resulted in a flash point of 223.53 °C, virtually identical to the pure used oil, whereas the highest value was observed for colloidal nanosilver (225.22 °C, an increase of 1.7 °C relative to the control). In all tested samples − both with and without additives − the flash point of used oils was higher than that of fresh oils, which reflects the typical thickening and oxidation processes during operation. The most pronounced temperature increase occurred in the sample with colloidal nanosilver. In contrast, the oil containing ceramic EM showed a flash point only 0.5 °C higher than that of the used oil without additives, indicating that this additive had a negligible effect on ignition temperature while maintaining its environmental and microbiological benefits.

Table 6 presents the flash point results for used oil along with basic statistical analysis.

**Table 6:** Flash point results for used oil with basic statistical analysis.

	Flash point of used oil		
	Value average	Standard deviation	*P*-value
UO	223.53	0.57	–
UO + EM(F)	224.47	0.64	0.13
UO + EM(C)	223.53	0.57	1.00
UO + SS	224.53	0.47	0.10
UO + CN	225.22	0.47	0.03

The statistical analysis presented in Table 6 confirms that the addition of the tested substances did not lower the flash point of the used engine oil, which indicates that they do not impair its thermal properties. In most cases, the values were similar to those of the control sample (oil without additives), confirming their neutral effect on this parameter. Moreover, in the case of colloidal nanosilver additive, a statistically significant increase in the flash point was observed, which may indicate an additional beneficial effect of this additive.

### Water content in diesel fuel

The chemical composition of mineral oil includes an oil base obtained by refining crude oil and a set of additives designed to improve the properties of the oil base. Synthetic oil also uses an oil base and additives, but this base consists of organic compounds produced synthetically through chemical synthesis processes.

In both cases, the presence of water is undesirable − it leads to a deterioration in the properties of the lubricant, accelerates its degradation and can adversely affect the operation of equipment. The exceptions are flame-retardant fluids and water-oil emulsions, in which the presence of water is natural and intentional. The immiscibility of oil and water results directly from differences in their molecular structure. Water molecules are polar, while oil molecules are non-polar. Polarity is a property of chemical compounds consisting in the uneven distribution of electrical charges in a molecule.

In a water molecule, the electrons of the hydrogen atoms are shifted towards the oxygen atom, which causes a partial positive charge on the hydrogen side and a partial negative charge on the oxygen side. This phenomenon is referred to as bond polarisation^44^.

However, the final impact on polarity is also determined by the geometry of the molecule: even if individual bonds are polarised, the molecule may be non-polar if its structure is symmetrical (e.g. ‘linear’). Oil molecules have this structure, which makes them non-polar. According to the principle of ‘like dissolves like’, polar substances dissolve well in other polar substances, and non-polar substances dissolve well in non-polar substances. For this reason, oil and water do not mix with each other due to differences in polarity^45^.

The results of water content determinations are presented in the form of graphs in the figures below. They show an analysis of the effect of individual additives on the water level in engine oil samples. Each sample − both pure oil and oil with additives − was tested three times under identical conditions. The article presents the average values obtained for each of the cases studied.

**Fig. 10:**
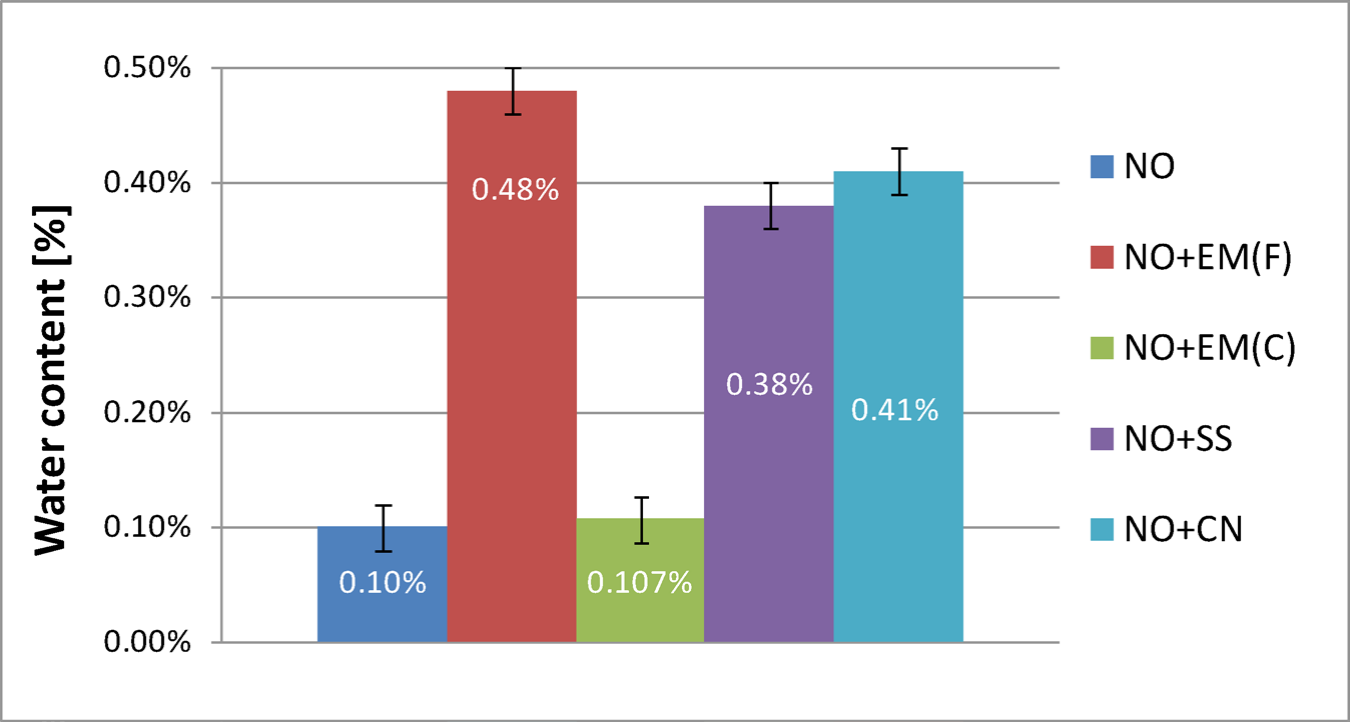
Water content of pure new engine oil and engine oil with additives in a concentration of 2%.

The analysis of the results presented in Fig. 10 shows that the water content in the base engine oil without additives was 0.10%. The introduction of 2% additives caused varying effects depending on the formulation. The addition of liquid effective microorganisms (EM) increased the water content to 0.48%, representing an approximately fourfold rise compared with the control sample. In contrast, the use of ceramic EM had a negligible effect on this parameter, with a measured value of 0.107%, nearly identical to that of the base oil. For the silver-based additives, moderate increases in water content were observed. The silver solution yielded a value of 0.38%, while colloidal nanosilver produced 0.41%, corresponding to roughly three- to fourfold higher values than those recorded for the oil without additives. These results indicate that the increase in water content primarily reflects the aqueous nature of the tested additives rather than degradation of the oil itself.

All additives, except for effective microorganisms in ceramic form, caused an increase in water content compared to pure lubricating oil, which can be explained by the fact that most of them consist largely of water. The highest water content was recorded in the sample with liquid effective microorganisms, while the lowest − within the statistical error − was recorded in the sample with the addition of ceramic effective microorganisms.

On this basis, it can be concluded that the most beneficial additive in terms of minimising water content are effective microorganisms in ceramic form, as they do not introduce additional water into the system. However, this does not mean that other additives have a clearly negative effect on the properties of the oil, as their action is based on the formation of a water-oil emulsion in which water molecules (which are polar in nature) are dispersed in a non-polar oil phase. This type of stable emulsion can only be formed in the presence of surfactants, which enable the temporary combination of phases with different polarities.

Depending on the method used to obtain silver particles, different types of stabilisers are used. Among the stabilisers and protective agents used, PVP (polyvinylpyrrolidone) is the most commonly used. PVP is a homopolymer whose polyvinyl backbone contains polar groups in which N and O atoms have a strong affinity for silver ions and nanoparticles, causing PVP molecules to cover the surface of nanoparticles, preventing the formation of larger particles. As can be seen in Fig. 4, the additives mixed with oil formed emulsions, i.e. a mixture of oil, water and a surfactant. The creation of such an emulsion was possible because this mixture contains both a surfactant and an emulsifier. Surfactants adsorb at the boundary between oil and water, thereby reducing surface tension. Since silver solutions are formed by combining finely divided silver with protein, water or gelatin, it is gelatin that acts as a surfactant. In addition, the emulsifier stabilises the emulsions and coats the droplets in the emulsion, preventing them from combining or coalescing, and polyvinylpyrrolidone may be used for this purpose.

This means that despite such a high water content in the oil, it is not dangerous, because the added silver solution with a surfactant and stabiliser allows water to be absorbed up to a level of 1%, and this value was not reached in the tested samples.

Table 7 presents the water content results for used oil together with a basic statistical analysis.

**Table 7:** Water content results for new oil with basic statistical analysis.

	Water content of new oil		
	Value average (%)	Standard deviation (%)	*P*-value
NO	0.10	0.0159	–
NO + EM(F)	0.48	0.0365	**2.71 × 10** ^**− 8**^
NO + EM(C)	0.10	0.0138	0.7864
NO + SS	0.38	0.0415	**8.58 × 10** ^**− 7**^
NO + CN	0.41	0.0750	**2.04 × 10** ^**− 5**^

The statistical analysis presented in Table 7 indicates that only the ceramic EM(C) additive did not cause a statistically significant increase in the water content of the oil (*p* = 0.7864), maintaining a level comparable to that of the fresh oil (0.10 ± 0.0138%). For all other additives, the observed increase in water content was statistically significant (*p* < 0.001), with the highest value recorded for liquid EM(F) (0.48 ± 0.0365%). These results demonstrate that EM(C) had a neutral effect on the water content of the oil, whereas additives with higher inherent moisture content − particularly EM(F) − led to a measurable and statistically significant increase. This trend suggests that the water introduced together with certain biological additives may influence the physicochemical stability of the lubricant and should be carefully considered in practical applications.

**Fig. 11:**
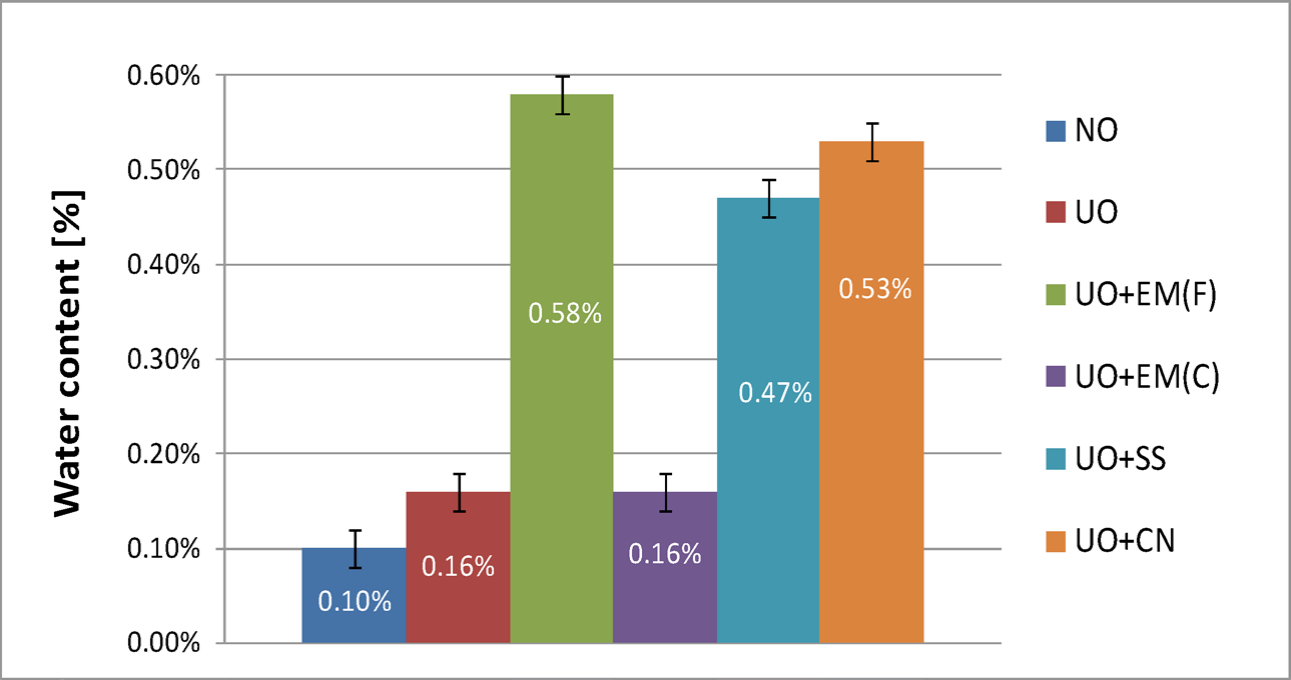
Water content of pure used engine oil and engine oil with additives in a concentration of 2%.

Figure 11 shows the water content in engine oil used without any additives and with additives. For comparison, the graph also shows the water content in clean, new lubricating oil. The tests used used oil, which, before being used in a marine engine, was mixed with the aforementioned additives in an amount representing 2% of the total oil volume.

As noted earlier, the water content in the fresh engine oil was 0.10%, while in the used oil without additives it increased to 0.16%, representing a 60% rise due to normal operation. The addition of liquid effective microorganisms (EM(F)) resulted in the highest measured water content, reaching 0.58%, which corresponds to an approximately fivefold increase compared with the fresh oil and more than threefold relative to the used oil. In contrast, the ceramic EM(C) additive showed no measurable effect on this parameter, maintaining the same value as the clean used oil (0.16%). The silver-based additives caused moderate increases in water content: 0.47% for the silver solution (SS) and 0.53% for colloidal nanosilver (CN). These results indicate that the observed differences mainly reflect the inherent water content of the additives themselves rather than degradation of the oil.

The observed trend of increasing water content in used oil with additives is similar to that obtained for new oil with additives, but the values are correspondingly higher, which results from oil overuse and deterioration of its operating parameters. In this case, too, the highest water content was found for liquid effective microorganisms, and the lowest for EM in ceramic form. The water content in samples with the addition of silver solution and colloidal silver was similar.

In terms of water content, the same result as for pure used lubricating oil was obtained for EM in the form of ceramics, which indicates their most neutral effect on this parameter and suggests that they are the most suitable additive to engine oil in the context of maintaining low water content.

Table 8 presents the water content results for used oil, together with a basic statistical analysis.

**Table 8:** Water content results for used oil with basic statistical analysis.

	Water content of new oil		
	Value average (%)	Standard deviation (%)	*P*-value
UO	0.16	0.034	–
UO + EM(F)	0.58	0.041	**1.22 × 10** ^**− 7**^
UO + EM(C)	0.16	0.017	0.8254
UO + SS	0.47	0.034	**5.30 × 10** ^**− 8**^
UO + CN	0.53	0.025	**5.95 × 10** ^**− 7**^

The analysis of the data presented in Table 8 showed that only the ceramic EM(C) additive did not cause a statistically significant increase in the water content of the used oil (*p* = 0.8254), maintaining a level comparable to that of the reference sample (0.16 ± 0.017% vs. 0.16 ± 0.034%). For all other additives, the increase in water content was statistically significant (*p* < 0.000001), with the highest value observed for liquid EM(F) (0.58 ± 0.041%). These findings indicate that EM(C) had a neutral effect on the water content of the oil, whereas additives with higher inherent water content − particularly EM(F) − led to a measurable and statistically significant increase. The observed trend suggests that excessive water introduction through certain biological additives may negatively influence the lubricating and oxidative stability of the oil during prolonged use.

### Acid and base number of lubricating oil

Ampholytes (amphiprotic substances) are chemical compounds whose molecules contain both acidic and basic groups. Lubricating oils contain both acidic and alkaline substances. In addition, some additives used to improve the performance of the oil may also exhibit both acidic and basic characteristics.

For this reason, oil can be considered an amphoteric substance which, depending on the environment, can behave as an acid or a base. Consequently, in order to track changes in oil during its use, it is necessary to monitor both its acidity, determined by the acid number (TAN), and its alkalinity, expressed as the base number (TBN).

**Fig. 12:**
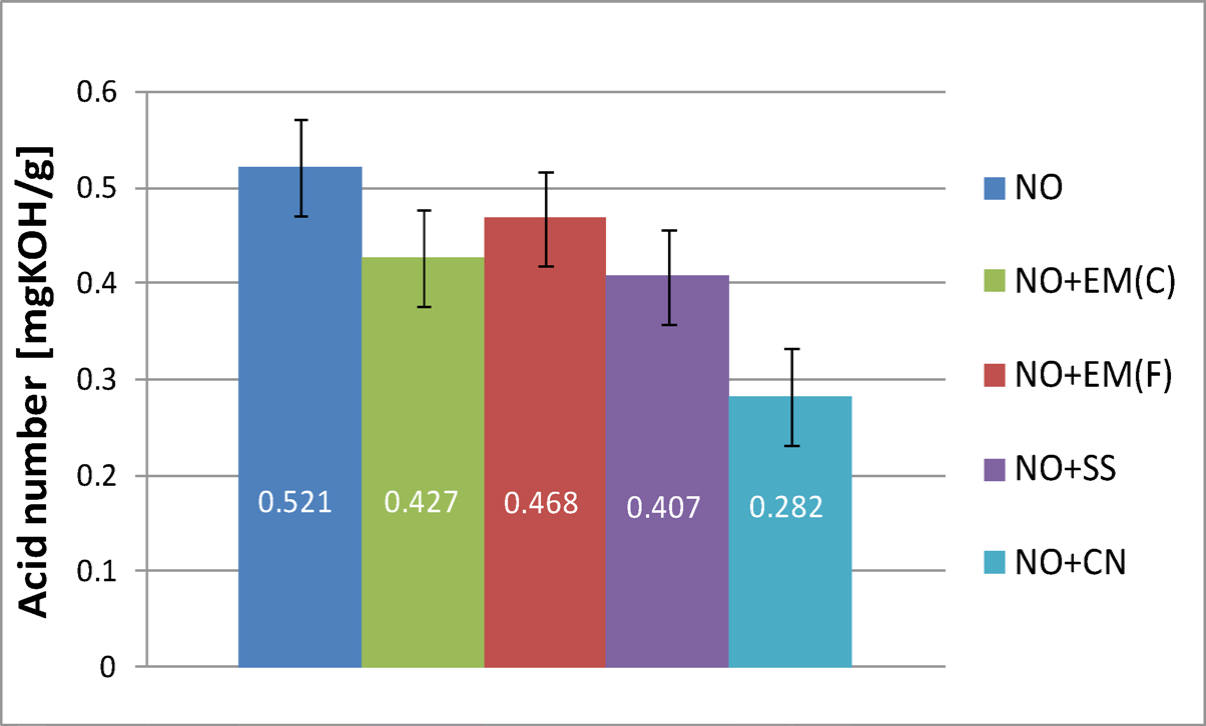
Acid number of pure new engine oil and engine oil with additives in a concentration of 2%.

**Fig. 13:**
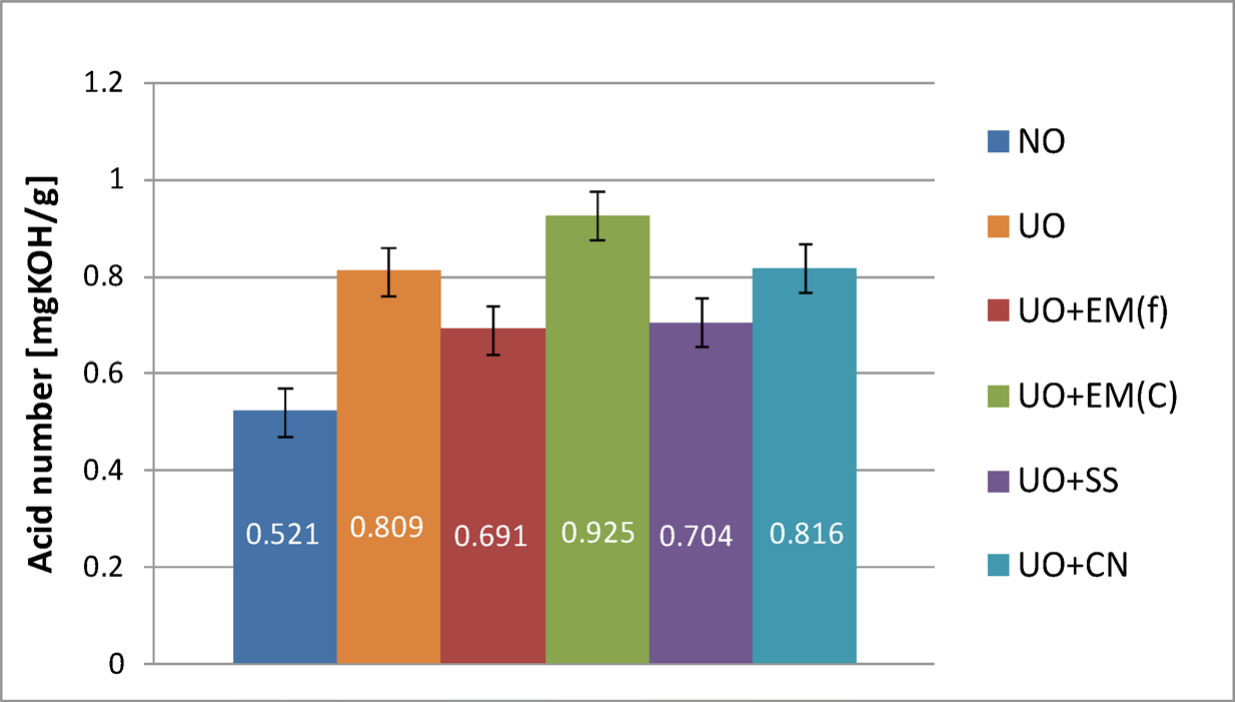
Acid number of pure used engine oil and engine oil with additives in a concentration of 2%.

**Fig. 14:**
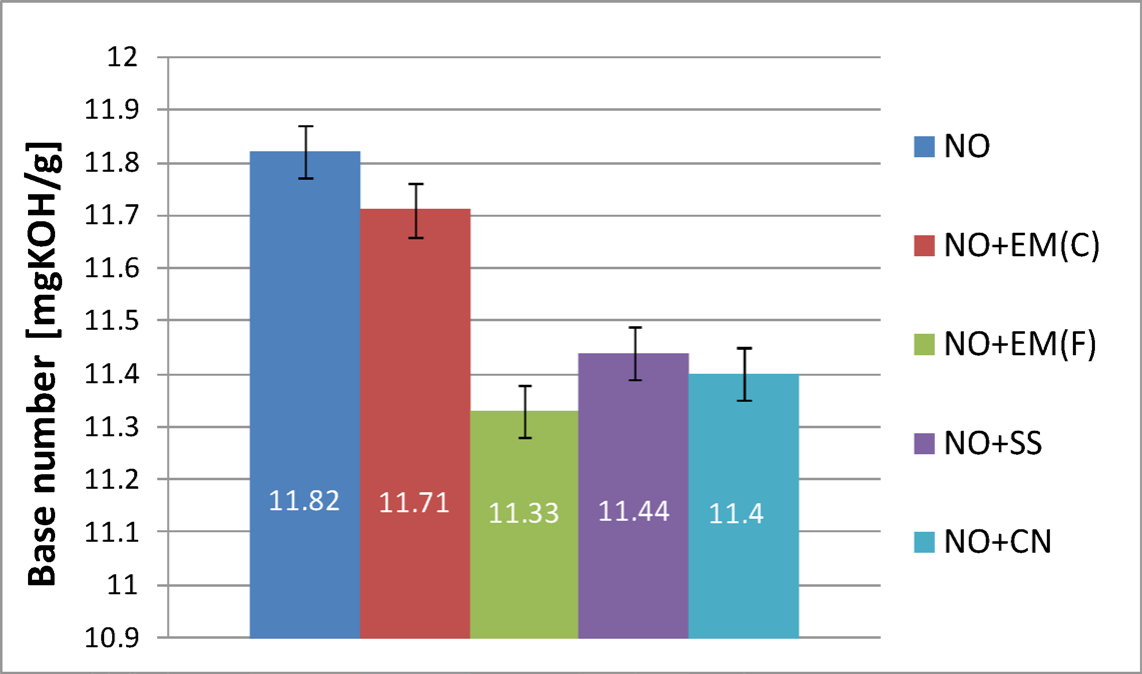
Base number of pure new engine oil and engine oil with additives in a concentration of 2%.

**Fig. 15:**
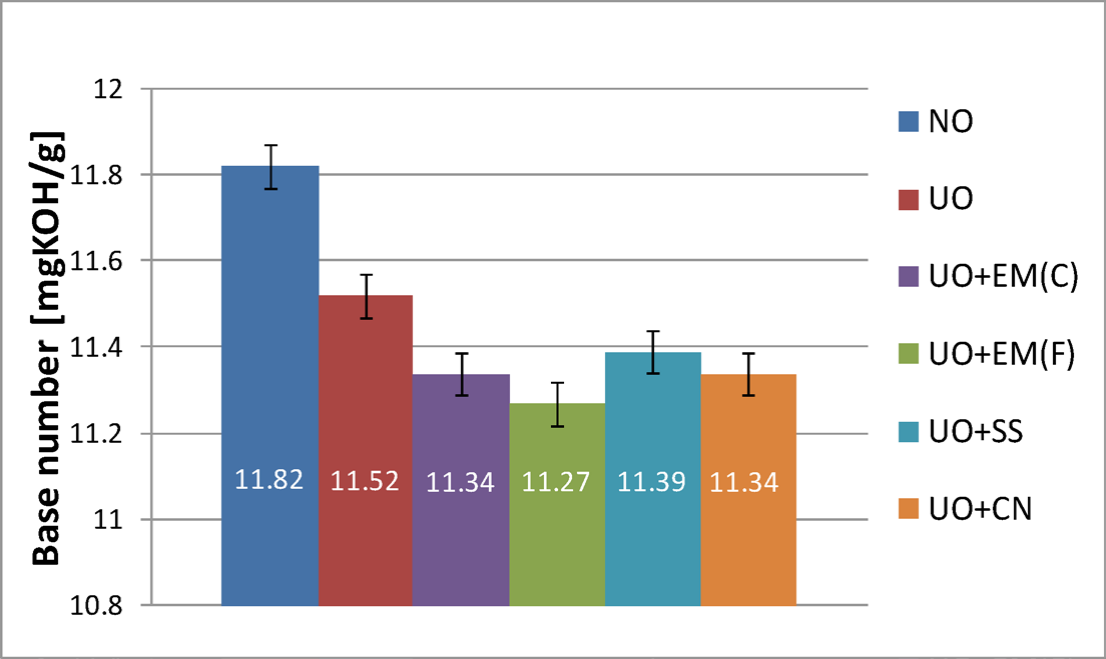
Base number of pure used engine oil and engine oil with additives in a concentration of 2%.

The results of the acid number (TAN) and base number (TBN) tests are presented in the form of graphs in the Figs. 12, 13, 14 and 15. These graphs illustrate the impact of individual additives on the acid number, which is a parameter used to assess the condition of engine oil, i.e. its degree of ageing, and on the base number, which determines the oil’s ability to neutralise acids generated during operation. These parameters are among the key indicators for assessing the quality of engine oils.

During engine operation, oil oxidation and fuel combustion processes occur, resulting in by-products, including acidic compounds. Each sample — both pure oil and oil with additives — was tested three times, and this study presents the average values obtained for each case. Figure 12 shows the acid number of new pure engine oil and engine oil with EM(F), EM(C) and a 2% solution of silver (SS) and colloidal nanosilver (CN).

Analysis of the results obtained showed that the acid number (TAN) of fresh engine oil is 0.521 mgKOH/g. The addition of 2% by volume of effective microorganisms (EM) reduced the acid number to 0.427 mgKOH/g for ceramic EM and to 0.468 mgKOH/g for liquid EM, which is the highest value among the additives tested. For ionic silver, a value of 0.407 mgKOH/g was obtained, similar to the result obtained for EM in ceramic form. The lowest acid number value was recorded after the use of colloidal nanosilver, which amounted to 0.282 mgKOH/g.

The acid number (TAN) is a measure of the concentration of acidic compounds in non-aqueous solutions and is one of the basic indicators for assessing the suitability of engine oil for further use. It is determined based on the amount of potassium hydroxide (KOH) needed to neutralise all acidic components contained in one gram of oil sample. An increase in TAN is usually the result of progressive oil oxidation, leading to the formation of acidic by-products. It may also indicate the depletion of antioxidant additives, which in turn promotes corrosion of the internal components of the device. This process is accelerated under conditions of elevated oil operating temperature. For this reason, the acid number should remain as low as possible, as excessive acidification of the oil can lead to premature damage to the system in operation. In all analysed cases, the use of additives resulted in a decrease in TAN, with the largest decrease observed after the addition of colloidal nanosilver. However, it is crucial to assess how the additives used affect the oil parameters after a certain period of operation in a marine engine.

Table 9 presents the acid number results for used oil along with basic statistical analysis.

**Table 9:** Acid number results for new oil with basic statistical analysis.

	Acid number of new oil		
	Value average	Standard deviation	*P*-value
NO	0.521	0.032	–
NO + EM(F)	0.427	0.048	0.1577
NO + EM(C)	0.468	0.027	0.1087
NO + SS	0.407	0.033	0.070
NO + CN	0.282	0.098	0.040

Table 9 shows that in the case of new oil, a tendency to reduce the acid number was observed after the addition of all the tested additives, which may indicate their beneficial stabilizing effect. The strongest effect was observed for the CN additive (0.282 ± 0.098), for which the decrease was statistically significant (*p* = 0.040), while for the other additives the changes did not reach statistical significance (*p* > 0.05). The results suggest that none of the additives used has a negative effect on the acid value of new oil.

In order to analyze the total acid number (TAN) in used oil—both without additives and with various additives—the measurement results are summarized in Fig. 13. To enable the assessment of changes occurring during operation, the graph also shows the TAN value obtained for clean, new engine oil.

The data presented in Fig. 13 indicate that the acid number (TAN) of the used engine oil without additives increased to 0.809 mgKOH/g, representing an approximately 55% rise compared with the fresh oil (0.521 mgKOH/g). The addition of effective microorganisms (EM) to the fresh oil led to further increases in TAN after use: by approximately 50% for the ceramic EM(C) and by over 95% for the liquid EM(F) relative to their corresponding fresh-oil values.

Although in both cases (oil without additives and oil with EM in ceramics) a similar 50% increase in acidity was recorded after processing, the use of EM in ceramic form proved to be more beneficial, as it reduced the initial acid number in fresh oil. A similar effect of initial TAN reduction was also observed for liquid EM, but in this case, the additive caused the highest increase in acid number during operation, which may accelerate oil degradation.

The addition of ionic silver produced an effect similar to that of EM in ceramics—after use, a TAN value of 0.704 mgKOH/g was obtained (an increase of approx. 50% compared to new oil), which means less acidification compared to oil without additives. In turn, colloidal nanosilver caused the largest increase in acid number to 0.816 mgKOH/g, even though the initial TAN value was the lowest for this additive.

In summary, the analysis of the results indicates that, in terms of acid number stabilization during operation, the most beneficial additives are EM in ceramic form and ionic silver solution.

Table 10 shows the acid water numbers for used oil along with basic statistical analysis.

**Table 10:** Acid number results for the oil used with basic statistical analysis.

	Acid number of used oil		
	Value average	Standard deviation	*P*-value
UO	0.809	0.025	–
UO + EM(F)	0.699	0.078	0.1595
UO + EM(C)	0.925	0.085	0.1808
UO + SS	0.704	0.071	0.1638
UO + CN	0.816	0.058	0.8917

Based on Table 10, it can be concluded that in the case of used oil, the acid number did not undergo statistically significant changes after the addition of any of the tested additives (*p* > 0.05), which indicates that they do not adversely affect this parameter during oil operation. Only a slight decrease in value is visible after the use of EM(F) and SS additives (0.699 and 0.704, respectively), which, however, was not statistically significant. This means that all tested additives are safe in terms of their impact on the acid number of used oil.

The results presented in Fig. 14 show that the base number (TBN) of the fresh engine oil without additives was 11.82 mgKOH/g. The addition of ceramic effective microorganisms (EM(C)) resulted in a slight decrease to 11.71 mgKOH/g, corresponding to a reduction of approximately 0.9% compared with the base oil. The liquid EM(F) caused the largest decline, lowering the TBN to 11.33 mgKOH/g (4.1% decrease). The silver-based additives also slightly reduced the base number, with values of 11.44 mgKOH/g for the silver solution (SS) and 11.40 mgKOH/g for colloidal nanosilver (CN), representing reductions of about 3.2% and 3.6%, respectively. Overall, all tested additives produced minor decreases in alkalinity, remaining within the typical experimental variation for lubricating oils.

Comparing the values obtained, it can be concluded that ceramic effective microorganisms have the least negative impact on the base number, making them the most beneficial additive among those tested if the goal is to preserve the alkaline properties of the oil.

The total base number (TBN) is a very important parameter because it characterizes the oil’s ability to neutralize acids formed during its use. This property is particularly associated with engine oils, where the aim is to neutralize acidic combustion products. Consequently, oil used in engines should have a specific level of alkalinity.

The higher the TBN value, the greater the oil’s ability to neutralize acids during engine operation, which protects the metal components of the system from corrosion. Over time and with vehicle use, the TBN gradually decreases as a result of the consumption of the additives contained in the oil that are responsible for maintaining its alkalinity.

Table 11 presents the results of the base number for used oil together with a basic statistical analysis.

**Table 11:** Base number results for new oil with basic statistical analysis.

	Base number of new oil		
	Value average	Standard deviation	*P*-value
NO	11.82	1.305	–
NO + EM(F)	11.71	1.305	0.6464
NO + EM(C)	11.33	0.465	0.8962
NO + SS	11.44	1.630	0.7662
NO + CN	11.40	1.046	0.7574

Table 11 shows that adding the tested substances to fresh oil did not cause statistically significant changes in the base number (*p* > 0.05 for all additives), which means that none of them has a negative effect on this parameter. The observed differences in mean values (from 11.33 to 11.71 mgKOH/g compared to 11.82 mgKOH/g for the base oil) are within the standard deviation, confirming the stability of the oil’s properties. These results suggest that all tested additives can be safely used without reducing the base number of fresh oil.

Figure 15 shows how individual additives mixed with fresh oil reduced the base number of used oil.

Figure 15 shows that the total base number (TBN) of the used engine oil without additives decreased from 11.82 mgKOH/g (fresh oil) to 11.52 mgKOH/g, corresponding to a 2.5% reduction. The addition of ceramic effective microorganisms (EM(C)) led to a slightly greater decrease to 11.34 mgKOH/g (4.1% decrease), while the liquid form (EM(F)) produced the largest reduction, lowering the TBN to 11.27 mgKOH/g (4.7% decrease).

The use of silver-based additives resulted in comparable values: 11.39 mgKOH/g for the silver solution (SS) and 11.34 mgKOH/g for colloidal nanosilver (CN). Among all tested samples, the oil containing silver solution retained the highest TBN value after engine operation, suggesting a slightly better ability to maintain the alkaline reserve of the oil under operating conditions.

In addition, Table 12 shows the flash point results for used oil along with basic statistical analysis.

**Table 12:** Base number results for used oil with basic statistical analysis.

	Base number of used oil		
	Value average	Standard deviation	*P*-value
UO	11.52	1.650	=
UO + EM(F)	11.27	1.645	0.8430
UO + EM(C)	11.34	1.645	0.9127
UO + SS	11.39	1.095	0.8947
UO + CN	11.34	1.395	0.8742

After analyzing the data in Table 12, it can be concluded that adding the tested substances to the used oil did not cause statistically significant changes in the base number (*p* > 0.05 for all additives), which means that none of them negatively affects the oil’s ability to neutralize acids. The average values (11.27–11.39 mgKOH/g) are very close to those for oil without additives (11.52 mgKOH/g) and fall within the standard deviation, which indicates the stability of this parameter. These results suggest that additives can be used without the risk of accelerated loss of base number during oil operation in the engine.

### Testing the kinematic viscosity and density of engine oil

The graphs below illustrate the effect of individual additives on the kinematic viscosity of engine oil samples. The analyses were carried out in the temperature range from 20 °C to 53 °C, i.e., up to the maximum value achievable using a heater connected to a viscometer. Next, using an interpolation procedure in accordance with ASTM D3446, the kinematic viscosity values at 100 °C were determined.

In order to observe viscosity changes over a wider temperature range, the tables list the oil parameters for temperatures of 40 °C and 100 °C. In addition, the density curve for fresh oil in the same temperature range is presented, which allowed for a more accurate determination of the kinematic viscosity.

**Fig. 16:**
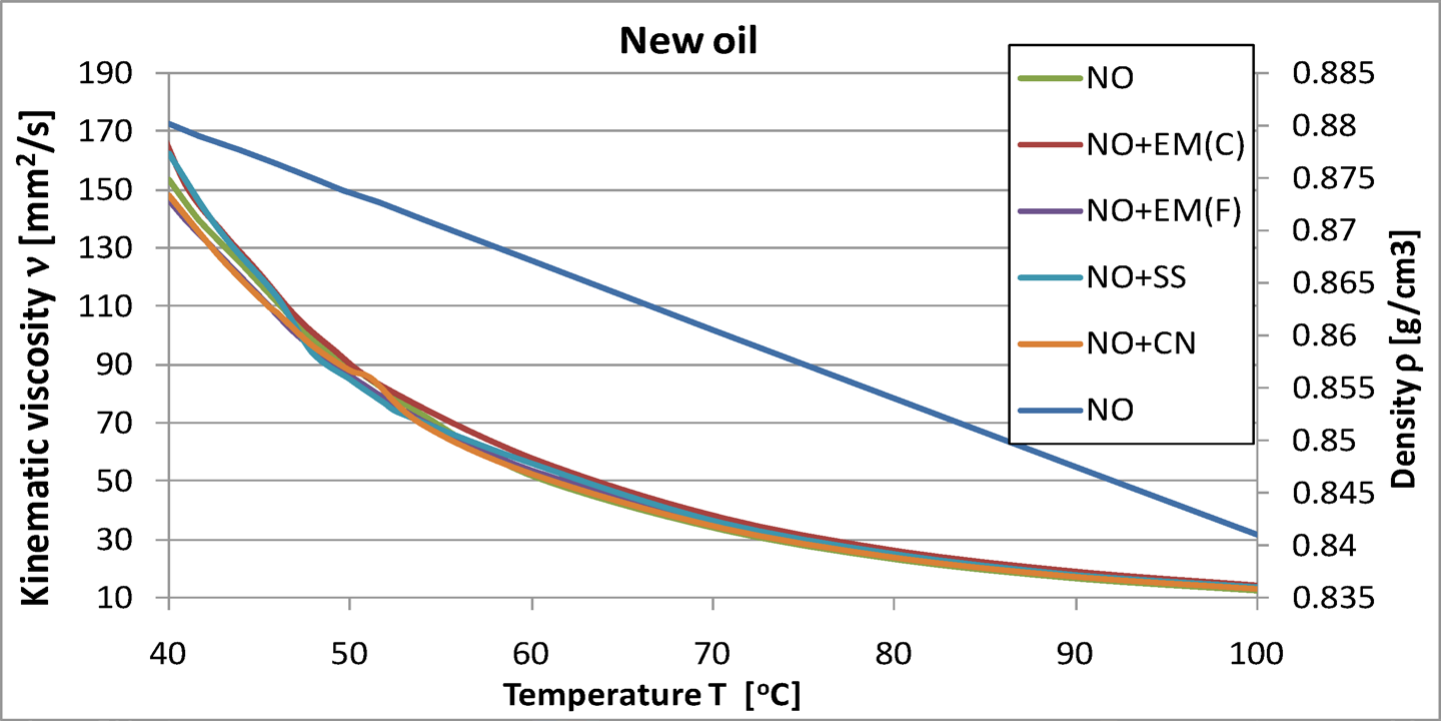
Kinematic viscosity and density of pure engine oil and engine oil with additives in a concentration of 2%.

Additionally, in order to increase the transparency of the data presentation, the test results are summarized in Tables 13 and 14. They also include values obtained at a temperature of − 10 °C, which allowed us to illustrate the oil parameters in sub-zero conditions.

**Table 13:** Comparison of kinematic viscosity of new oil without and with additives.

	Oil kinematic viscosity at -10 °C [mm^2^/s]	Oil kinematic viscosity at 40 °C [mm^2^/s]	Oil kinematic viscosity at 100 °C [mm^2^/s]
NO	17210.71	153.37	12.61
NO + EM(F)	9862.79	144.10	13.87
NO + EM(C)	13473.02	161.75	14.36
NO + SS	16123.52	162.44	13.68
NO + CN	12489.01	147.84	13.29

The analysis of the results presented in Fig. 16 shows that the kinematic viscosity of the base engine oil was 153.37 mm²/s at 40 °C and 12.61 mm²/s at 100 °C. The addition of effective microorganisms (EM) and silver-based additives produced comparable but distinct changes in viscosity profiles.

At 40 °C, samples containing ceramic EM(C) and silver solution (SS) exhibited slightly higher viscosity values − 161.75 mm²/s and 162.44 mm²/s, respectively − representing increases of approximately 5–6% compared with the base oil. In contrast, oils supplemented with liquid EM(F) and colloidal nanosilver (CN) displayed reduced viscosities of 144.10 mm²/s (6% decrease) and 147.84 mm²/s (4% decrease), respectively. These variations remained within the typical range observed for lubricating oils under similar testing conditions.

At 100 °C, the viscosity of the base oil remained at 12.61 mm²/s. Slightly higher values were obtained for EM-containing samples − 14.36 mm²/s for EM(C) and 13.87 mm²/s for EM(F) **−** corresponding to increases of approximately 14% and 10%. For the silver-based additives, the measured viscosities were 13.68 mm²/s for SS and 13.29 mm²/s for CN, which correspond to moderate increases of 8% and 5%, respectively. The relatively stable viscosity values across all samples suggest that the tested additives did not negatively affect the thermal-thinning behavior of the oil at elevated temperature.

Since the viscosity of engine oils at low temperature is critical for ensuring proper lubrication during cold starts, additional measurements were performed at − 10 °C. Under these conditions, the viscosity of the base oil was 17210.71 mm²/s. The lowest value was recorded for the sample containing liquid EM(F) − 9862.79 mm²/s − representing a decrease of approximately 43% relative to the base oil. For the remaining additives, the following values were obtained: 13473.02 mm²/s for ceramic EM(C), 12489.01 mm²/s for colloidal nanosilver (CN), and a value closest to the base oil for the silver solution (SS).

Overall, the observed viscosity variations across the tested temperature range (–10 °C to 100 °C) remained moderate and consistent with the normal operational limits of lubricating oils. The results demonstrate that none of the tested additives caused adverse changes in viscosity that could compromise the oil’s flow characteristics or engine protection capacity.

Using oil with too high viscosity at a given temperature can cause flow difficulties, leading to engine starting problems, increased fuel consumption, and excessive wear on structural components. On the other hand, oil with too low viscosity may not provide adequate lubrication (failure to generate proper oil pressure), resulting in similar operational problems. Considering that the viscosity values obtained for the tested additives did not differ significantly (approx. ±5%) from the results for the base oil, it can be concluded that their use does not have a negative impact on this parameter.

**Fig. 17:**
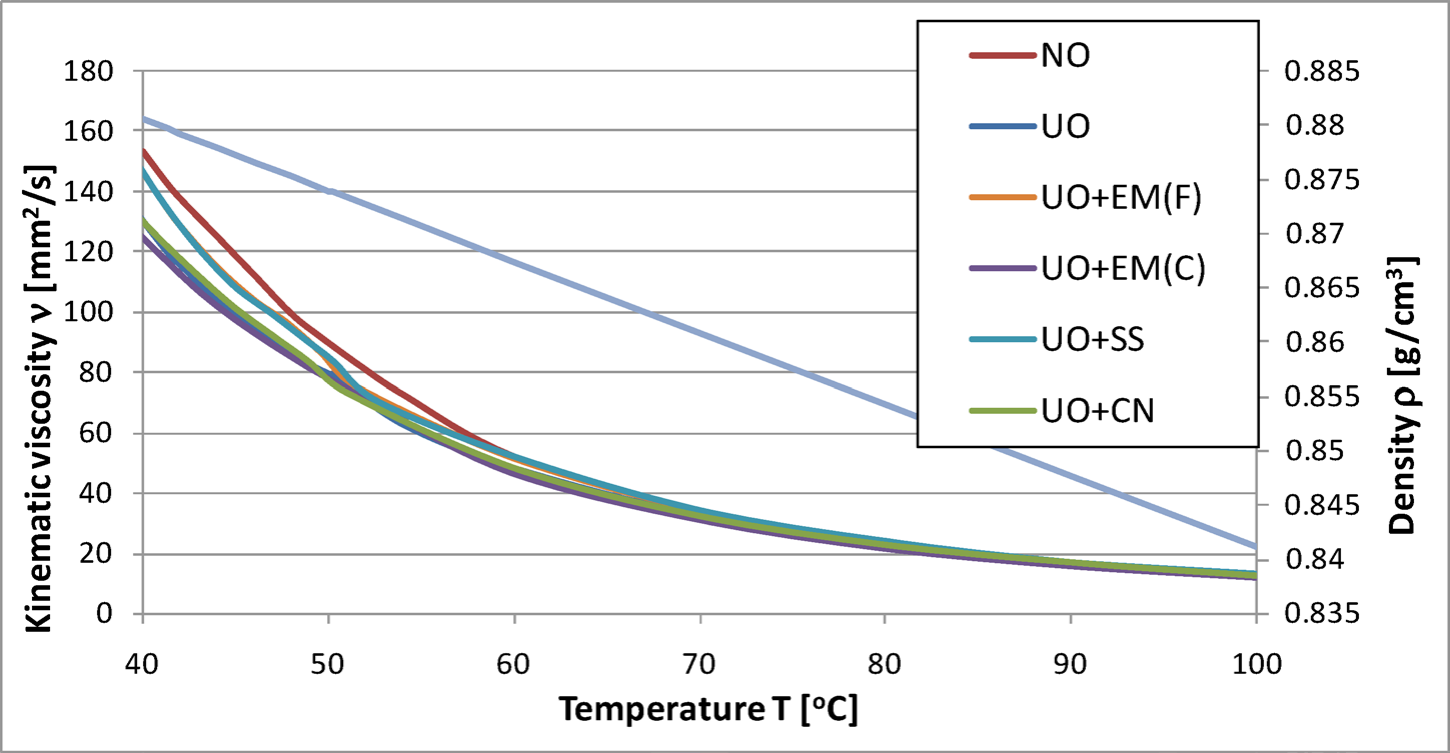
Kinematic viscosity and density of pure used engine oil and engine oil with additives in a concentration of 2%.

**Table 14:** Comparison of kinematic viscosity of used oil without and with various additives.

	Oil kinematic viscosity at -10 °C [mm^2^/s]	Oil kinematic viscosity at 40 °C [mm^2^/s]	Oil kinematic viscosity at 100 °C [mm^2^/s]
NO	17210.71	153.37	12.61
UO	7655.47	124.31	12.69
UO + EM(F)	7397.98	123.16	12.19
UO + EM(C)	13319.10	146.50	12.91
UO + SS	8096.30	129.21	12.84
UO + CC	15567.52	153.95	13.03

The results presented in Fig. 17 show that the kinematic viscosity of the used engine oil without additives was 124.31 mm²/s at 40 °C and 12.69 mm²/s at 100 °C, compared with 153.37 mm²/s and 12.61 mm²/s, respectively, for the fresh oil. This represents a 19% decrease in viscosity at 40 °C and a negligible 0.6% increase at 100 °C, which is consistent with the expected shear and thermal degradation occurring during engine operation.

The addition of liquid effective microorganisms EM(F) and colloidal nanosilver (CN) yielded viscosity values very similar to those of the used oil without additives. At 40 °C, the viscosities were 123.16 mm²/s and 129.21 mm²/s, respectively, corresponding to decreases of 20% and 16% relative to the fresh oil. At 100 °C, the values were 12.69 mm²/s for EM(F) and 12.84 mm²/s for CN, nearly identical to the used oil and differing by less than 2% from the base sample.

In contrast, the addition of ceramic EM(C) and silver solution (SS) resulted in viscosity values at 40 °C of 146.50 mm²/s and 153.96 mm²/s, respectively − closely matching those of the fresh oil (differences below 5%). At 100 °C, the viscosities were 12.91 mm²/s for EM(C) and 13.03 mm²/s for SS, both characteristic of stable performance at the engine’s normal operating temperature. These results suggest that EM(C) and SS contribute to maintaining the oil’s viscosity stability under thermal and mechanical stress.

Because engines often operate under sub-zero conditions, additional tests were conducted at − 10°C. At this temperature, the kinematic viscosity of the base oil was 17,210.71 mm²/s. The most comparable values were obtained for ceramic EM (13319.10 mm²/s) and silver solution (15567.52 mm²/s), corresponding to decreases of approximately 23% and 10%, respectively, relative to the fresh oil. The lowest viscosities were recorded for oils containing liquid EM (7397.98 mm²/s, 57% decrease) and colloidal nanosilver (8096.30 mm²/s, 53% decrease).

Across all tested conditions, viscosity variations remained within acceptable operational limits, and no adverse effects of the additives on oil rheology were observed. The data indicate that ceramic EM and silver solution most effectively preserved the viscosity characteristics of the oil, while liquid EM and colloidal nanosilver slightly reduced viscosity, particularly at lower temperatures.

The observed results can be partly explained by the increase in viscosity after mixing new oil with additives. In the case of ceramic microorganisms and silver solution, the initial increase in viscosity translated into more stable values after the oil had been used in the engine, unlike water-based additives (EM in liquid form and colloidal nanosilver), which showed a more pronounced decrease in parameters.

In summary, additives in the form of silver solution and effective ceramic microorganisms had the most beneficial effect on maintaining stable viscosity. Their use allowed the lubricating oil properties to be maintained for longer compared to other variants tested.

## Conclusion

The aim of this study was to evaluate the effect of selected ecological additives – silver solution, colloidal nanosilver and effective microorganisms (EM) in liquid and ceramic form – on the physicochemical and microbiological properties of marine engine oil. These additives were used for the first time in lubricating oils for marine applications.

The analyses showed that the tested additives had diverse effects on the flash point and water content of the oil. Liquid EM and EM ceramics caused a measurable decrease in the flash point (to approximately 222 °C), whereas silver solution and colloidal nanosilver slightly increased this parameter (by about 1 °C) relative to the base oil. Although the observed reduction for liquid EM was moderate, it may indicate limited suitability of this additive for combustion engines due to potential ignition sensitivity.

Regarding water content, most additives − except for EM ceramics − increased the parameter, which can be attributed to their aqueous composition. Ceramic EM showed the lowest water content (0.16%), suggesting its potential as a more stable additive. All tested additives decreased the acid number, with colloidal nanosilver showing the strongest effect, while silver solution provided the most favorable balance between acid and base number stability.

The viscosity values remained within ± 5% of the reference oil, indicating that the applied additives did not adversely affect this key operational parameter. After simulated engine operation, oils with silver solution and ceramic EM maintained the most stable viscosity profile, which may prolong oil service life.

Microbiological analyses indicated that all additives reduced microbial contamination. Silver solution demonstrated the strongest antibacterial activity (approximately 40-fold reduction), whereas ceramic EM showed the highest antifungal efficiency (about 65-fold reduction). These findings suggest that silver solution and EM ceramics may offer a balanced improvement in both physicochemical and antimicrobial performance.

The results obtained provide a basis for further research into the long-term effectiveness of environmentally friendly additives in real operating conditions of marine engines. In subsequent stages of research, it is worth conducting long-term bench tests, including an assessment of the durability of additives, their impact on the tribological wear of engine components, and potential interactions with oil degradation products during operation.

It would also be valuable to investigate the synergistic effect between EM ceramics and silver at lower concentrations in order to optimise antimicrobial efficacy while reducing costs.

### Limitations of the study

During the study, the author encountered certain difficulties that may have affected the results obtained. The author undertook research into the use of these additives as environmentally friendly, as there are other areas where they are used successfully. These additives have a positive impact on environmental protection and combustion engine performance.

This research was conducted under laboratory conditions, which do not fully reflect the complex operating environment of an actual marine engine. The results should therefore be considered preliminary and require confirmation in long-term operational tests. Another limitation is the use of a single type of base oil and a fixed concentration of additives, which does not allow for the full range of possible interactions between additives and different oils to be captured.

In future studies, it would be worthwhile to expand the scope to include an assessment of tribological wear and an analysis of the impact of additives on oil oxidation and ageing processes.

In addition, it would also be necessary to investigate the mechanisms of action of additives, i.e. the combination of two or three additives that have the most beneficial effect on the properties of the tested engine oil. It is also important to investigate the impact of these additives on the technical condition of the combustion engine and the wear of its components.

Despite these limitations, the results obtained provide an important basis for the further development of environmentally friendly additives for lubricating oils used in marine engines.

## Data Availability

All data generated or analyzed during this study are included in this published article.
